# The efficacy and applicability of chimeric antigen receptor (CAR) T cell-based regimens for primary bone tumors: A comprehensive review of current evidence

**DOI:** 10.1016/j.jbo.2024.100635

**Published:** 2024-09-22

**Authors:** Atefeh Barzegari, Fateme Salemi, Amirhossein Kamyab, Adarsh Aratikatla, Negar Nejati, Mojgan Valizade, Ehab Eltouny, Alireza Ebrahimi

**Affiliations:** a Cell Science Research Center, Kian Immune Cell Co, Tehran, Iran; b Hematology, Oncology and Stem Cell Transplantation Research Center, Research Institute for Oncology, Hematology and Cell Therapy, Tehran University of Medical Sciences, Tehran, Iran; c Faculty of Medicine, Fasa University of Medical Sciences, Fasa, Iran; d School of Medicine, Royal College of Surgeons in Ireland, Dublin, County Dublin, Ireland; e Pediatric Cell and Gene Therapy Research Centre, Gene, Cell & Tissue Research Institute, Tehran University of Medical Sciences, Iran; f School of Medicine, Shahid Sadoughi University of Medical Sciences, Yazd, Iran; g Department of Orthopaedic Surgery, Massachusetts General Hospital, Harvard Medical School, Boston, MA, United States

**Keywords:** Primary bone tumors (PBT), Osteosarcoma (OS), Ewing’s sarcoma (ES), Chimeric Antigen Receptor (CAR), CAR T cell

## Abstract

Primary bone tumors (PBT), although rare, could pose significant mortality and morbidity risks due to their high incidence of lung metastasis. Survival rates of patients with PBTs may vary based on the tumor type, therapeutic interventions, and the time of diagnosis. Despite advances in the management of patients with these tumors over the past four decades, the survival rates seem not to have improved significantly, implicating the need for novel therapeutic interventions. Surgical resection with wide margins, radiotherapy, and systemic chemotherapy are the main lines of treatment for PBTs. Neoadjuvant and adjuvant chemotherapy, along with emerging immunotherapeutic approaches such as chimeric antigen receptor (CAR)-T cell therapy, have the potential to improve the treatment outcomes for patients with PBTs. CAR-T cell therapy has been introduced as an option in hematologic malignancies, with FDA approval for several CD19-targeting CAR-T cell products. This review aims to highlight the potential of immunotherapeutic strategies, specifically CAR T cell therapy, in managing PBTs.

## Highlights

• Primary bone tumors (PBTs) have a low incidence, but cause significant patient burden.
• CAR T-cell therapy has been used in treating hematological malignancies.
• CAR T-cells may be used in solid tumors to improve survival in patients with PBTs.
• This review summarizes findings on CAR T-cell therapy for PBTs.

## Graphical abstract

**Figure:**
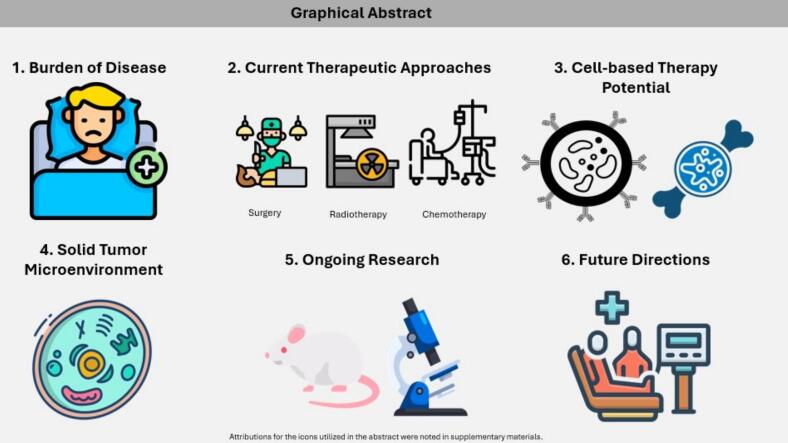

## 1 Introduction

### 1.1 Burden of primary bone tumors

Primary bone tumors (PBT) are estimated to account for nearly 0.2 % of all malignant tumors; however, the true incidence is difficult to determine due to the rarity of these tumors [1]. PBTs have an age-specific distribution; chondrosarcoma is the most common PBT among the adult population, while osteosarcoma and Ewing sarcoma are more commonly seen in children and adolescents [2], [3], [4]. Despite the low prevalence and incidence rate of these tumors, the associated mortality and morbidity are extremely high, mainly due to the high risk of lung metastasis in osteosarcomas and Ewing's sarcomas [1], [5].

Osteosarcoma, Ewing's sarcoma, and chondrosarcoma are estimated to have around 53.9 %, 50.6 %, and 75.2 % 5-year survival, respectively [6]. This chance of survival might decrease to as low as 20 % in the presence of metastasis [7]. Nearly 30 % of patients with PBT are at risk of developing relapse during the five years post-operatively, with the most commonly reported sites of recurrence reported to be lung and bone metastases [8], [9], [10]. Despite significant advances in screening and diagnosis processes, the survival rates of patients with PBT appeared to remain the same during the past forty years [1], [11], [12], highlighting the critical need for novel therapeutic interventions [7].

Surgical resection, radiotherapy, and systemic therapy constitute the principal modalities for the treatment of primary bone tumors. Enbloc resection with wide margins is the preferred primary therapy for resectable osseous lesions [13], [14], [15]. Multiple analyses have demonstrated that achieving negative histological margins correlates with prolonged disease-free survival (DFS) exceeding 5 years postoperatively [16], [17]. Adjuvant radiotherapy may be utilized to enhance local control and DFS when combined with surgery, while definitive radiotherapy alone could be considered for surgically unresectable tumors [18], [19]. Neoadjuvant chemotherapy is often used to downstage high-grade bone tumors before resection, whereas adjuvant chemotherapy may be warranted following excision if positive margins are encountered [20]. Standard multi-agent chemotherapy protocols incorporating doxorubicin, cisplatin, cyclophosphamide, and high-dose methotrexate are among the main options [21], [22]. Emerging immunotherapeutic-based regimens have also exhibited potential in the treatment of PBTs.

Scientists have been trying to utilize different immunotherapeutic treatments for several types of hematological malignancies and solid tumors, including PBTs, over the past decades [23]. This approach to fighting against cancer cells could be categorized into different methodologies, including, but not limited to, immune checkpoint inhibitors (ICIs), tumor vaccines, oncolytic virotherapy, and adoptive cell transfer (ACT). One of the strategies that has shown potential, particularly in hematological malignancies, is the use of ACT utilizing chimeric antigen receptor (CAR) engineered T-cells able to detect tumor-associated/specific antigens (TAAs/TSAs) and mount potent anti-neoplastic responses [19], [24], [25].

### 1.2 Current indications of CAR T cell therapy

CAR T cell therapy has been approved to use in lymphoma, leukemia, and multiple myeloma [26]. Food and Drug Administration (FDA) has authorized six total CAR T-cell products, four of which target cluster of differentiation 19 (CD19) including Tisagenlecleucel for refractory adult lymphoma, Axicabtagene ciloleucel for adult non-Hodgkin lymphoma and diffuse large B cell lymphoma, Brexucabtagene autoleucel for refractory mantle cell lymphoma and adult B-cell acute lymphoblastic leukemia, and lisocabtagene maraleucel for adult large B cell lymphoma [27], [28], [29], [30]; two non-CD19 CAR T cells have also been indicated in multiple myeloma, including Ciltacabtagene autoleucel and Idecabtagene vicleucel [27], [31], [32].

### 1.3 Generations of CAR T cells

CAR-T cell therapy, a variation of ACT in which T cells are designed to target TSAs or TAAs to bypass human leukocyte antigen restrictions, has different types of designs [1]. A CAR is a synthetically engineered receptor containing an extracellular antigen recognition domain, made of a single-chain variable fragment (scFv) with variable light (VL) and heavy (VH) chains made from one monoclonal antibody, one transmembrane domain, and intracellular signaling domain(s) originating from the T cell receptor CD3-z chain, that can interact with co-stimulatory molecules such as CD28 or 41BB to activate T cells [33], [34], [35], [36]. In contrast to conventional T cells that require attachment of the T cell receptor (TCR) to peptide-major histocompatibility complex (pMHC) ligands for activation, CAR-engineered T cells incorporate extracellular scFv, conferring antigen specificity in an MHC-unrestricted manner, allowing CAR T cells to recognize and elicit cytotoxicity against tumor cells expressing the TSA/TAAs [1], [37]. Following adoptive transfer, CAR-engineered T cells demonstrate potent proliferation and can establish long-term memory with sustained cytotoxic functionality against malignant cells displaying the antigens [38].

**Fig. 1:**
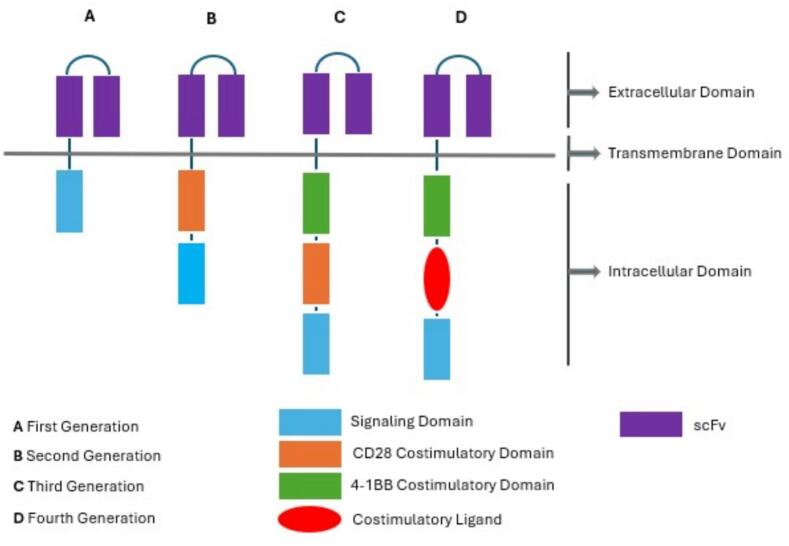
Different generations of CAR T cells.

The first generation of CARs contains only a TCR-derived CD3 constant signaling domain that is incapable of maintaining stability on the T cell membrane, resulting in poor persistence and efficacy [39]. Second and third generations incorporated one or more co-stimulatory signaling domains like CD28 or 4-1BB for full activation and survival signals, dramatically improving CAR T cell expansion, persistence, and anti-tumor role [40]. Currently, second-generation CARs are the most common format used clinically [40]. Fourth-generation CAR T cells, known as T cells redirected for universal cytokine killing (TRUCKs), enhance second- and third-generation platforms by incorporating a construct that enables the inducible production of proinflammatory cytokines such as IL-12, IL-15, IL-18, or IL-21 [39]. TRUCKs, delivering a substantial amount of pro-inflammatory cytokines to the tumor location, stimulate T cells and recruit immunologic cells to the tumor microenvironment (TME) (Fig. 1) [37], [41], [42]. Fifth-generation CARs have the same frame as those of the 2nd generation as well as a shorter cytoplasmic IL-2 receptor chain domain, which links to the transcription factor STAT3, enabling immunosuppressive-resistant CAR T cells [43].

### 1.4 Obstacles to broader application of CAR T cell therapy

While promising, broader clinical applications of CAR T cell therapy encounter significant challenges that have so far limited its utility. The clinical success of CAR T cell treatments has been constrained by adverse events, such as on-target, off-tumor toxicity arising from the recognition of healthy tissue expressing the target antigen [44]. CAR T cell activation and an excessive immunological response might create a condition known as cytokine release syndrome (CRS), known by significantly high levels of inflammatory cytokines [45], [46]. Additional toxicities of CAR T cell therapy are immune effector cell-associated neurotoxicity syndrome (ICANS), defined by cerebral edema and encephalopathy, as well as delayed immune-mediated effects such as hemophagocytic lymphohistiocytosis (HLH), resulting from unconstrained activation the T cells [47]. While CD19-directed CAR T cell therapy was outstandingly successful in hematologic cancers, outcomes in solid tumors have been more limited, attributed to multiple challenges, including paucity of TSAs, intratumoral and intertumoral antigen heterogeneity and antigen loss, defective migration and tumor invasion of the T cells, immunosuppressive networks in the TME comprising cytokines, immunosuppressive cell subsets, and physical barriers impeding access to tumor cells [48], [49], [50]. Further enhancements in suicide gene platforms, cytokine-signaling disruption, and CAR T cell engineering are imperative to improve therapeutic indices. To overcome TME-imposed limitations, next-generation armored CAR T cells co-expressing immunostimulatory cytokines and fourth-generation CARs incorporating custom costimulatory domains have been developed [24], [51]. Local delivery of the T cells and employment of oncolytic viruses encoding chemotactic factors to enhance tumor-homing are also under evaluation [24], [25], [52]. Continued refinement of case models recapitulating the TME, together with efforts to enhance CAR T cell engineering, represent critical priorities to achieve meaningful clinical efficacy against solid tumors.

### 1.5 Study objective

CAR T cell therapy is a promising immunotherapy for targeting neoplastic cells in PBTs, enhancing anti-tumor immunity, and potentially extending patient survival. Various TSAs and TAAs have been identified as potential targets for CAR T cell approaches in PBTs, such as VEGFR2, IGF1R, ROR1, GD2, IL-11 receptor chain, B7-H3 (CD276), EphA2, attIL-12, HER2, ALP-1, and NKG2D ligands (Table 1) [44], [53], [54], [55], [56], [57], [58], [59], [60], [61], [62], [63], [64], [65], [66], [67], [68], [69], [70], [71], [72], [73], [74], [75], [76], [77], [78]. CAR T cells targeting TAAs could directly kill PBT cells expressing these antigens and prevent metastasis by recognizing disseminated cancer cells with the same antigens [7].While several studies have investigated CAR T cell therapy for bone malignancies, there is no comprehensive evaluation comparing findings across different target antigens and CAR constructs. This systematic review aimed to consolidate data and identify the most promising antigen and CAR construct combinations to optimize clinical outcomes, addressing the challenges of improving patient outcomes and managing disease burden [50], [79], [80].

**Table 1:** Characteristics of the included studies on the efficacy of chimeric antigen receptor (CAR) T-cell therapy in primary bone tumors.

Author, year, country	Type of cancer	Tumoral cell line	Target Ag (expression status)	CAR Gen	Co-stim domain	Combination therapy	T cell culture medium ILs
Mensali, 2023	OS	OSA, U2OS, SaOS-2, 143B, G-292, and MG-63, LM7	ALP-1	2nd	CD34	None	IL-2
Zhang, 2022, China	OS	HOS, U-2 OS, SW1353, Saos-2	B7-H3 (All the 4 cell lines of OS highly expressed the B7-H3 protein)	3rd	CD28 and 4-1BB	None	rh IL-2
Hu, 2022, US	OS	OS31, OS1, OS02, OS09, and OS60-SJ	CSV (30 %)	2nd	CD28	None	rhIL-2, rhIL-7, rhIL-15
Prinzing, 2021, US	OS	LM7	EphA2, HER2, or IL13Rα2	2nd	CD28ζ	None	IL-7, IL-15
Wiebel, 2021, Germany	OS	HOS, U-2 OS, and MG-63	GD2	2nd	BBζ	EZH2 inhibitor tazemetostat, brefeldin A, PKC modulators phorbol-12-myristat-13-acetate.	rh IL-2
Long, 2021, China	SBC	Tumor cells of the chordoma samples show high expression of B7-H3	B7-H3 (was the most frequent antigen being expressed in tumoral tissue)	2nd	4-1BB and CD3-z	None	IL-2
Park, 2021, US	OS	143B & U-2 OS	GD2/HER-2	2^nd^ (EAT)	BsAb^a^	None	IL-2, IL15Rα-IL-15
Talbot, 2021, US	OS	LM7.ffLuc	B7-H3 (82.3 %)	2nd	4-1BB and CD28	None	IL-7/IL-15
Altvater, 2021, Germany	ES	A4573, TC-71, and VH-64 A673, TC-32, THP-1	GD2 and HLA-G1	2nd	4-1BB	None	None
Lange, 2021, US	OS and ES	LM7 and A673	HER-2 and EphA2	2nd	CD28 and 4-1BB	None	IL-7/IL-15
Hsu, 2021, US	OS and ES	143B, U2OS, MG63, A673 and ES8/143b	EphA2 (All cell lines expressed EphA2 protein)	2nd	4-1BB	None	IL-7/IL-15
Park, 2020, US	OS	143b, OSOS1B, TEOSC1, HGSOS	GD2/HER2 (Most of OS cell lines highly expressed GD2 &/or HER2 Ag.)	2nd	NA	without and with PD-1 and PDL-1 inhibitors	None
Englisch, 2020, Germany	ES	293T, A204, and MS-EwS-16-GFP	VEGFR-2 (low to high levels of expression)	2nd	4-1BB	None	None
Chulanetra, 2020, China	OS	HOS & U2OS	GD2 (99 % and 80.1 %, respectively)	4th	4-1BB and CD28	sub-toxic level of doxorubicin	IL-2, IL-7, IL-15
Kailayangiri, 2019, Germany	ES	VH-64, RM-82, and WE-68, MS-EwS-1, 4, 6, 16, 15, 34, A4573, 5838, TTC-466, and TC-32, A673, TC-71, SK-ES-1, RD-ES, and CADO-ES-1, SK-N-MC	GD2	2nd	CD28	EZH2 Inhibition (GSK126)	None
Wang, 2019, China	OS	Human OS lines MNNG/HOS, U2OS, MG-63 and Saos-2	CD166 (significant expression (36.9 to 96.7 %) in OS tumor tissue compared with fibroblast cell line NIH/3 T3 adjacent cells).	2nd	4-1BB	None	IL-2, IL-15
Majzner, 2019, US	OS, ES	MG63.3 (OS), EW8 (ES)	B7-H3 (>90 % of pediatric sarcomas expressed B7-H3)	3rd	CD137 (4-1BB) and CD28	None	rh IL-2, rh IL-4
Chulanetra, 2020, China	OS	HOS & U2OS	GD2 (99 % and 80.1 %, respectively)	4th	4-1BB and CD28	sub-toxic level of doxorubicin	IL-2, IL-7, IL-15
Charan, 2019, US	ES	ES2, ES4, SKNEP1 & TC71 MAN020E	GD2	3rd	CD28 and OX40	HGF-targeted neutralizing antibody (AMG102)	IL-2
Fernandez, 2017, Spain	OS	531MII YFP-luc, MG-63, and U-2	NKG2DL (low to very high levels)	2nd	4-1BB	None	IL-2
Long, 2016, US	ES, OS^a^	OS: 143b, G292, and MG63. ES: EW8, RMS559, & RH30.	GD2 (20 % of ES & 100 % of OS expressed GD2)	3rd	CD28, OX40.ζ	without & with SC surgical implantation of ATRA pellets. Ctrl g received Sham surgeries	rh IL-2
Ahmed, 2015, US	OS	LM7	HER-2 (65.2 %)	2nd	CD28	None	IL-2
Huang, 2015, US	ES, OS	ES: A673, EWS502, Rh1, SKNMC, TC32, TC71; OS: OS17, U2OS, SaOS2	IGF1R, ROR1 (OS & ES cell lines broadly expressed IGF1R & ROR1)	2nd	4-1BB	None	IL-2, IL-7
Liebsch, 2013, Germany	ES	VH-64	GD2	2nd	CD28 and TCR z	None	IL-2
Kailayangiri, 2012, Germany	ES	TC-32, TC-71, A4573 and VH-64	GD2 (ranged between RFIs of 2.7 ± 2.1 & 52.6 ± 6.2)	2nd	CD28	None	None
Lehner, 2012 Germany	ES	STA ET-1, −2, −3, −6, −8.2, −10, −11, ER-ESFT-1, CADO-ES1, TC-71, WE-68, VH-64	NKG2D (NKG2D-Ls was broadly expressed by ESFT cell lines)	2nd	CD28	None	IL-2
Rainusso, 2012, US	TICs in OS	MNNG/HOS and 143B	HER2 (OS TICs were uniformly HER2+, and highly expressed HER2)	2nd	CD28	MTX (OS cells with sarcosphere potential (TICs) are resistant to MTX in vitro.)	None
Huang, 2011, US	OS	CCH-OS-D, SAOS2, & LM7, KRIB	IL-11Rα (58.2 %)	2nd	CD28	None	IL-2, IL-26
Ahmed, 2008, US	OS	LM7.eGFP. FFLuc	HER-2 (low expression)	2nd	CD28	None	Rh IL-2

Abbreviations: Ag: Antigen, ATRA: All-trans retinoic acid, att: Cell membrane-anchored and tumor-targeted, BsAb: Bispecific antibodies, CAR: chimeric antigen receptor, Co-stim: co-stimulatory, CSV:cell-surface vimentin, EZH2: enhancer of zeste homolog 2, EAT: ex vivo armed T cells, ES: Ewing sarcoma, ESFT: Ewing’s sarcoma family of tumors, Gen: Generation, IL: interleukin, IGF1R: type I insulin-like growth factor receptor, KO: knockout, MTX: methotrexate, NKG2DL: natural killer group 2 member D ligand, OS: osteosarcoma, PKC: protein kinase C, PD-1: Programmed Cell Death Protein 1, PDL-1: Programmed Cell Death Ligand 1, rh: recombinant human, RFI: relative fluorescence intensity, ROR1: tyrosine kinase-like orphan receptor 1, SC: subcutaneous, SBC: Skull base Chordoma, TIC: tumor-initiating cell, VGFR-2: Vascular endothelial growth factor receptor 2.

a T cells armed with IgG-[L]-scFv GD2-BsAb (GD2-EAT) exerted significant and durable antitumor responses. In comparison with HER2-IgG conjugate-armed T cells, HER2-EATs showed higher BsAb densities and a greater ability to kill tumor cells. In vivo, HER2-EATs were much more effective for tumor response and survival.

### 1.6 Search strategy

Our query was conducted across PubMed/MEDLINE, Scopus, Web of Science (ISI), and Google Scholar databases. Using medical subject headings (MESH) and non-MESH terms, the query was changed to match each database format without date restriction until November 2023 (Supplementary S1). Following the entry of each paper into the reference management tool and the removal of duplicates, the bibliographies were manually explored to find further applicable sources. The Preferred Reporting Items for Systematic Reviews and Meta-Analyses (PRISMA) statements were employed to present the results [81]. The study protocol was registered in the International Prospective Register of Systematic Reviews (PROSPERO) database (registration code: CRD42024507352).

### 1.7 Eligibility criteria

All studies evaluating the application of CAR T cells in PBTs were included in this systematic review. The entry requirements were to report the outcomes related to CAR T cell-based therapeutics, including efficacy, safety, and survival. The studies that focused on other ACT approaches, such as engineered natural killer cells and those reporting irrelevant outcomes, were excluded. Additionally, the publication type is limited to full-length articles, excluding systematic reviews, meta-analyses, letters, editorials, commentaries, conference abstracts, and case reports.

### 1.8 Data extraction

Two independent researchers (A.B. and F.S.) retrieved the first author's name, publication date, location, type of cancer, target antigen, CAR-T cell generation, co-stimulatory domain, combination therapy, and T cell culture medium interleukins. Specific assay results that evaluated cytotoxicity and cytokine secretion were extracted from in vitro studies. For in vivo studies, the following items were extracted: mouse strain, randomization, all models of experiment conduction, control group, type of tumor cell and cancer cell injection, number of injected tumor cells and cancer cells, tumor size before and after CAR-T cell therapy, cytotoxicity and cytokine release assays implicated, tumor infiltration and metastasis, and survival evaluation.

### 1.9 Quality assessment

Two authors (A.B. and N.N.) separately evaluated all the included papers and discussed the controversies with the third author (F.S. and A.E.). Animal studies were rated according to SYRCLE’s risk of bias [82]. Due to the lack of a standard quality assessment tool for in vitro studies, a checklist was designed based on Tran et al.’s systematic review and meta-analysis [83].

## 2 Results

### 2.1 Study characteristics

**Fig. 2:**
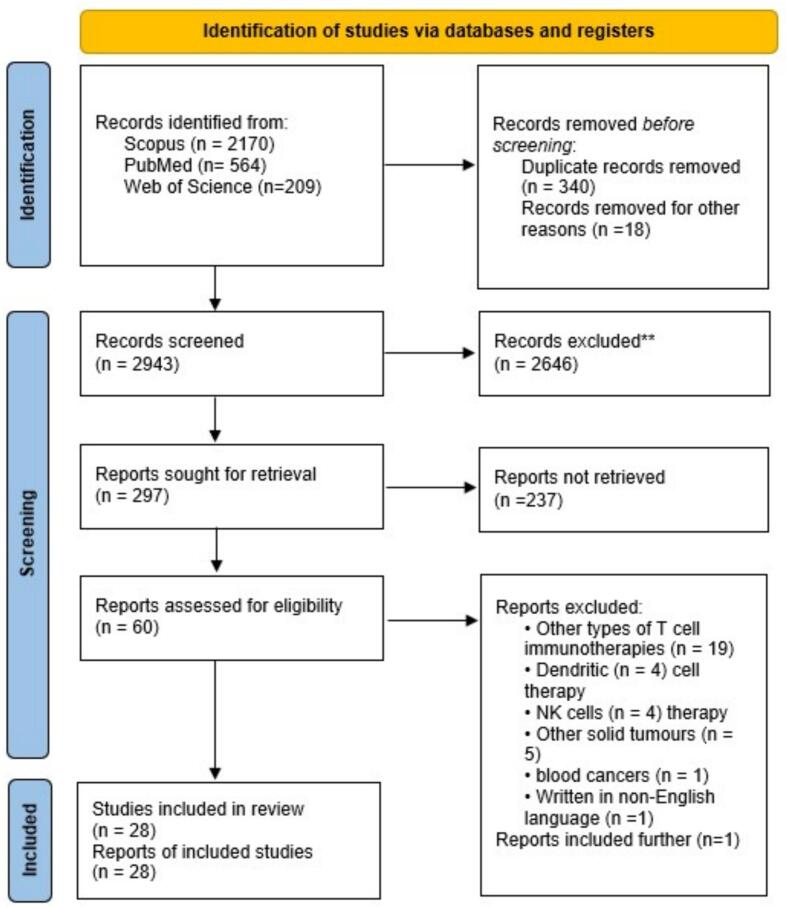
PRISMA flowchart of the study identification and selection process for the systematic review.

The initial scan yielded 3301 potentially relevant references, including 564 from PubMed, 2170 from Scopus, and 209 from Web of Science (Fig. 2). Following the deletion of 358 duplicates, the titles and abstracts of the residual 2943 cases were evaluated for relevance to PBT and CAR T cell therapy. This filtering process led to 297 articles for full-text review. Studies were excluded if they examined non-CAR T cell immunotherapies (n = 19) [68], [84], [85], [86], [87], [88], [89], [90], [91], [92], [93], [94], [95], [96], [97], [98], [99], [100], [101], dendritic cell (n = 4) [102], [103], [104], [105] or natural killer cell therapies (n = 5) [106], [107], [108], [109], non-osseous solid tumors (n = 3) [91], [105], [110], [111], [112], hematological malignancies (n = 1) [113], or were non-English language publications (n = 1) [114]. Twenty-eight papers had the qualifying requirements and were incorporated into this study. Of the 28 eligible studies, 16 were performed both in vitro and in vivo experiments [56], [60], [61], [63], [64], [65], [68], [69], [70], [71], [73], [75], [78], [115], [116], 7 were performed in vitro [58], [59], [62], [66], [67], [76], [117], 4 were in vivo animal studies [55], [57], [74], [77], and one was a clinical trial [72].

To date, the only phase I/II clinical trial (NCT00902044) evaluating the efficacy of CAR T cell therapy in eradication of PBT has utilized HER-2 as target antigen [72]. Nineteen patients with recurrent/refractory HER2-positive osteosarcoma (n = 16) or Ewing sarcoma (n = 1) received escalating doses (1 × 10^4^–1 × 10^8^ cells/m^2^) of HER2-targeted CD28-containing CAR T cells [72]. Fourteen of 16 patients administered ≥1 × 10^5^ HER2-CAR T cells demonstrated detectable peripheral blood levels for at least 3 h post-infusion [72]. Furthermore, durable HER2-CAR T cell engraftment (>6 weeks) was observed in 7 of 9 evaluable patients receiving ≥1 × 10^6^ cells [72]. HER2-CAR T infiltration into tumor sites was confirmed in two resected lesions. Of evaluable cases, 23 % experienced stable disease for 3–14 months, including one patient with 90 % tumor necrosis after surgical resection. The median survival rate across all treated individuals was 10.3 months (range 5.1–29.1 months) [72].

Table 1, Table 2, Table 3 provide an overview of the qualifying studies' attributes. They were published from 2008 to 2023 in the United States (n = 14) [56], [57], [60], [61], [63], [64], [65], [70], [72], [73], [77], [78], [115], [116], Germany (n = 8) [58], [62], [67], [74], [75], [76], [77], [117], China (n = 4) [55], [59], [66], [100], Norway (n = 1) [53] and Spain (n = 1) [71].

**Table 2:** The findings of in vitro studies conducted to evaluate the efficacy of CAR T-cell therapy in primary bone tumors.

Author, year	Mechanism of action	Main result
Hu, 2022	**Cytotoxicity assay:**1.5 × 10^6^ tumor cells + 1.5 × 10^6^ T cells (E:T ratio: 1:1) E1: ttIL12 CAR T cells. C1: B7H3 CAR T C2:wtIL12	**Cytotoxicity assay:** Despite C T cells, E1 T cells produced more perforin against tumor cells and these increases were significantly reduced by blockade of CSV, confirming E1 bind to CSV Ag and activate against CSV^+^ tumor cells.
Mensali, 2023	**Cytotoxicity assay:** 1 × 10^4^ tumor cells + T cells (5:1 E:T ratios) E1: OSCAR-1 E2: OSCAR-3 C: mock T cells (at 10:1) **CAR T cell killing assay(bioluminescence-based):** 3 × 10^5^ tumor cells + CAR T cells (at 10:1 E:T ratio)	Both OSCAR constructs were able to redirect T cells against these targets and caused significant cell death of primary OS cells at different E:T ratios.
Wiebel, 2021	**Cytotoxicity assay:** 10^4^ tumor cells + GD2 CAR T cells (E:T ratio: 40:1 to 10:1) C: tumor cells alone	**Cytotoxicity assay:** Higher tumor cell confluencies was significantly associated with GD2 expression, and increased sensitivity of tumor cell targets to GD2 CAR T cell-mediated in vitro cytolysis^a^.
Long, 2021	**Cytotoxicity assay:** tumor cells + B7-H3 CAR T cells (E:T ratio: 2:1). E1:B7-H3^+^ tumor cells E2: B7-H3^-^ tumor cells C: UTD T cells.	**Cytotoxicity assay:** B7-H3 CAR T cells in E1 significantly inhibited tumoral cell growth and tumor sphere formation by secreting granzyme-B and perforin as well as IFN-γ and TNF-α, and up-regulating activation markers (CD69 and CD25).
Talbot, 2021	**Cytotoxicity assay (Impedance-based):** 3 × 10^4^ tumor cells + 1.5 × 10^5^ B7-H3 CAR T cells (E:T ratio: 1:1 or alone). C1: Tumor cells alone C2: Tumor cells in DMSO Rechallenge: 3 times **Cytokine assay:** Tumor cells + T cells (E:T ratio: 1:1 or alone). Production of IFN- γ and IL2 was measured. **Antigen-Stimulated Expansion Assay:** Tumor cells + T cells (E:T ratio: 1:1 or alone). After 4 days T cells were counted and fold change from baseline was calculated.	**Cytotoxicity assay:** B7-H3 CAR T cells rapidly killed OS cells, (>95 % cytolysis), **Cytokine assay:** B7-H3 CAR T cells secreted significantly higher levels of IFN- γ and IL-2 compared to C1, C2. No significant cytokine synthesis was found by CAR T cells without tumor cells, confirming that CAR recognition of tumor cells caused cytokine production. **Antigen-Stimulated Expansion Assay:** B7-H3-CAR T cells also expanded in the presence of tumor cells in contrast to C1, C2. This expansion was antigen specific since no significant difference was observed in the absence of tumor cells between B7-H3- and Ctrl-CAR T cell.
Altvater, 2021	**Cytokine assay:** 1 × 10^5^ irradiated HLA-G1-transduced tumor cells b + 1 × 10^5^ GD2CAR T cells. C: mock T cells. **CD107a assay:** ES cells + GD2 CAR T cells (E:T ratio: 1:1) in the presence of CD107a BV510. **Cytotoxicity assay (calcein acetyoxymethyl release assay):** ES cells + GD2 CAR T cells (E:T ratio of 40:1 or alone).	Despite myeloid bystander cells, the presence of HLA-G1 on the cell surface of GD2-positive ES cells did not significantly reduce the function of GD2CART. In response to coincubation with HLA-G1 and HLA-E GD2^+^ ES cell lines, GD2 CAR T cells upregulated CD107a. HLA-G1 and HLA-E on ES cells did not affect GD2 CAR T cell cytolysis or cytokine release. Despite their expression in ES under inflammatory settings, non-classical HLA molecules have little effect on CAR T cells, hence it is uncertain if combining CAR T cell therapy with HLA-G or HLA-E checkpoint inhibition may improve ES management.
Lange, 2021	**Repeat Stimulation and cytokine assay:** 5 × 10^5^ (or 1 × 10^5^) ES cells + 1 × 10^6^ (or 5 × 10^5^) T-cells. IL-15 added to HER2-CAR T cells, Cytokines were then quantified. E1: EphA2-CAR E2:EphA2-CAR.GM18 c T-cell E3: HER2-CAR T cells **Cytotoxicity assay (MTS assay):** 1.9 × 10^4^ ES cells + serial dilutions of CAR T cells. E1: HER2-CAR T cells C: EphA2-ΔCAR T cells	In this method which mimics chronic Ag, E2 showed greater expansion and cytokine production compared with unmodified counterparts targeting EphA2 or HER2. E2 T-cells produced higher levels of cytokines and this only reached statistical significance for IL-13.
Park, 2021	**Cytotoxicity assay**: EAT over a range of E:T ratios and BsAb arming dosages was studied E1:GD2-EATs E2: HER2-EATs.	The maximum cytotoxicity was quantitated between 500 and 20,000 BsAb molecules per T cell for both E1 and E2. E1 and E2 had lower potencies (EC50) than soluble BsAbs under identical E:T ratios; however, the maximal killing efficacy was comparable.
Park, 2020	**51Cr release assay (Antibody-dependent T cell-mediated cytotoxicity):** activated T cells + OS cells (10:1 E:T ratio). Cytotoxicity was compared to Ctrl BsAb with T cells and each BsAb alone.	GD2-BsAb-armed T cells (GD2-EATs) and HER2-BsAb-armed T cells (HER2-EATs) showed stable BsAb binding and effective cytotoxicity against OS cell lines over a range of E:T ratios and antibody doses.
Hsu, 2020	**Cytotoxicity assay (Impedance-based tumour cell killing assay):** EphA2 + OS / ES cells and murine NIH3T3 cells (negative control: C3) + T cells **Cytokine assay:** EphA2 + OS / ES cells + T cells. Supernatants were then removed and analyzed by ELISA for IFN-γ and IL-2. E: EphA2- CAR T cells (E:T ratios: 1:1, 1:5 and 1:25), C1: UTD T cells (E:T ratio: 1:1), C2: control ΔCAR T cells (E:T ratio: 1:1)	**Cytotoxicity assay:** incubation of E cells with target cells caused significant cytotoxicity at E:T ratios of between 1:1 and 1:27. No cytotoxic effect was seen after coculture of tumor cells with C1 and C2 group or after co-culture of C3 cells with E group. **Cytokine assay:** significantly higher levels of cytokine secretion by E group over levels produced by control cells, indicative of antigen-specific tumour cell recognition by E group.
Englisch, 2020	**Cytotoxicity Assay** **1. AM release assay:** ES cells + T cells (E:T ratio: 40:1 to 10:1 or alone) specific lysis was calculated^a^. **2. Sequential killing assay:** ES cells + T cells (E:T ratio: 1:2), then cells were counted by flow cytometry using a CD45-Ab. **Spheroid assay:** to more closely mimic cellular interactions in vitro than by 2D cultures, a spheroid killing assay was performed. E1: Short hinge huVEGFR2 CAR T cell, E2: Medium hinge huVEGFR2 CAR T cell, E3: Long hinge huVEGFR2 CAR T cell, C1: UTD T cell, C2: CD19 CAR T cell	**AM release assay:** stimulation of E groups with target cells induced expression of the degranulation marker CD107a, IFN-γ and TNF-α compared to C groups. Higher proportions of CD107a-positive cells were found among E1 and E2 groups compared to E3 group. **Sequential killing assay:** all E groups repeatedly lysed target cells over three consecutive rounds and the capacity for sequential target cell lysis did not differ significantly among them. **Spheroid assay:** all E groups lysed spheroids generated from target ES cells. E1 and E2 had stronger activity against spheroids than E3. Despite E1, E2 and E3 groups exerted nonspecific activity against VEGFR2-negative spheroids.
Kailayangiri, 2019	**Cytokine Assay:** 5 × 10^5^ GD2^−^ ES cells + 5 × 10^5^ T cells. **CD107a Degranulation Assay**: 3 × 10^5^ ES+3 × 10^5^ T cells. E1: EZH2 inhibitor (GSK126) treated ES cells + GD2 CAR T cells. C1: untreated ES cells + GD2 CAR T cells. C2: GD2 CAR T cells alone. **Cytotoxicity Assay (AM release assay):** E:10^5^ GSK126 treated GD2^−^ES cells + GD2 CAR T cells (E:T ratios: 40:1 to 10:1). C1: untreated ES cells + GD2 CAR T cells. C2: UTD T cells.	**Cytokine Assay and CD107a Degranulation Assay:** In E group ES cells significantly secreted TNF-α, IFN-γ, and CD107 despite C1 and C2 group. **Cytotoxicity Assay:** GSK126 sensitizes GD2 ^−^ ES cells to antigen-specific functional interactions with GD2 CAR T cells and to CAR T cell in vitro cytolysis.
Wang, 2019	**Cytotoxicity assay (LDH release Assay):** OS cells or CD166^−^ NIH/3 T3 cells (negative control tumor cells) + T cells (E:T ratios: 20:1, 10:1 and 1:1). **Cytokine assay**: OS cells + 10^6^ T cells (E:T ratio: 1:1). IL-2, IL-4, IL-6, IL-10, TNF-α, and IFN-γ release was measured using the cytometric bead array. E: CD166 CAR T cells C: UTD T cells.	**Cytotoxicity assay:** E group efficiently lysed CD166high tumor cells, but not negative control and CD166low target cells, which confirmed the association between E group cytotoxicity with the level of CD166 expression. Besides, the cytotoxicity of E cells against OS cells was accompanied with the elevated E:T ratio. **Cytokine assay**: despite C group, high levels of TNF-α, IFN-γ, IL-4, IL-6 and IL-10 was released by E group that was associated with the quantity of CD166 expression.
Chulanetra, 2019	**Cytotoxicity assay of GD2 CAR T:** 3 × 10^4^ OS cells + GD2 CAR T cells (E:T ratios: 4:1, 2:1, 1:1 and 1:2). C1: glypican 3 (GPC3)-CART cells, C2: CD19-CAR T cells **Cytotoxicity assay of Chemotherapy (MTT assay):** 10^5^ OS cells + 54.0–3.8 µM carboplatin, 8,000–500 µM ifosfamide, 2.0–0.125 µM etoposide, and 0.25–0.0156 µM DXR. **Cytotoxicity assay of Chemotherapy and GD2 CAR T cells (Combination therapy):** 3 × 10^4^ Chemotherapy-treated OS cells d + GD2 CAR T cells (E:T ratio: 1:2). C: CD19-CAR T cells.	**Cytotoxicity assay of GD2 CAR:** CAR T cells not only efficiently killed various types of tumor cells, but also could kill target tumors for extended period. **MTT assay**: The IC50s of carboplatin, ifosfamide, etoposide and doxorubicin on osteosarcoma were determined to be 10.39 μM, 1,202 μM, 0.51 μM and 0.102 μM, respectively. **Combination therapy:** Only DXR pretreatment synergized with GD2 CAR T cells cytotoxicity against OS cells (decreased tumor cell viability and PD-L1 intensity, and increased caspase 3/7 activity).
Charan, 2019	**Cytokine assay:** 5 × 10^5^ irradiated ES cells + 5 × 10^5^ GD2 CAR T cells (E:T ratio: 1:1) **Cytotoxicity assay (51Cr-release assay):** 1 × 10^4^ tumor cells + 5 × 10^4^ GD2 CAR T cells C: UTD T cells.	GD2 CAR T cells showed significant cytotoxicity against GD2^+^ ES cells; and caused increased IFN-γ, IL-2, and TNF-α production by GD2^+^ ES cell lines.
Fernandez, 2017	**Cytotoxicity assay (traditional 4-h europium-TDA release assay):** OS cells + T cells (E:T ratios: 20:1, 10:1, 5:1, and 2.5:1). **Colony-forming assay:** CD34^+^ healthy cell lines were + CAR T cells at 20:1, 10:1, 5:1, and 1:1 E:T ratios, or cultured alone (control). To rule out NKG2D CAR-T cells could cause hematologic toxicity. E: NKG2D CAR T cells, C: UTD CD45RA T cells.	**Cytotoxicity assay:** E cells showed significantly higher cytotoxic capacity against target cells compared with C cells at all E:T ratios. There was no apparent relation between NKG2DL expression and sensitivity to E cells' cytotoxicity. **Colony-forming assay:** CD34^+^ cells capacity to form colonies was unaffected after coculture with NKG2D-CAR redirected memory T cells, indicating no therapy-induced hematologic toxicity.
Long, 2016	**Cytotoxicity assay (51Cr release assay):** OS and ES cells + GD2 CAR T cells at varying E:T ratios^a^. **Cytokine assay:** OS and ES cells + GD2 CAR T cells at varying E:T ratios. G-CSF and IL-8 production by tumor cell lines was evaluated within supernatants by ELISA. E: GD2 CAR T cells C: UTD T cells.	**Cytotoxicity assay (51Cr release assay):** E group *efficiently lyse GD2*^+^*target cells.* Very little lysis of the GD2^−^ cells was observed, indicating high specificity of E group. Thus, it can be concluded that E group showed comparable efficacy against OS and ES cells versus NB cells. **Cytokine assay:** production of G-CSF and IL-8 in OS cells compared to NB cells against E group was observed.
Huang, 2015	**Cytotoxicity assay (51Cr release assay):** OS cells + T cells at varying E:T ratios (60:1, 20:1, and 6:1)^a^. **Cytokine assay:** 2 × 10^4^ OS and ES cells + 1 × 10^5^ T cells. Supernatants were assayed using IFN-γ or TNF-α ELISA kits. E1: IGF1R CAR T cells E: ROR1 CAR T cells C: UTD T cells	**Cytotoxicity assay:** despite C group, E1 group significantly killed IGF1R^+^ OS cells at all 3 E:T ratios compared to IGF1R^−^ control cells suggesting that E cells are highly specific in targeting IGF1R. Also, E2 group killed ROR1^+^ OS cells at E:T ratios of 20:1 and 6:1 compared to ROR1^−^ control cells suggesting that E1 and E2 groups are highly specific in targeting IGF1R and ROR1 respectively. **Cytokine assay:** compared to C group, Both E1 and E2 group produced high-levels of IL-13, IFN-γ and TNF-α in an antigen-specific manner.
Huang, 2012	**Cytotoxicity assay (51Cr release assay):** 5 × 10^3^ OS cells + T cells^a^. E: IL-11Ra-CAR T cells. C: UTD T cells.	E group had improved cytotoxicity against all OS cell lines compared to C group.
Kailayangiri, 2012	E: GD2 CAR T cells, C: UTD T cells **Cytotoxicity assay:** 2.5 × 10^4^ ES cells + 10^6^ T cells at various E:T ratios. **Cytokine assay:** **1.**10^6^ ES cells + 10^6^ T cells stained with IFN- γ and TNF-α specific Abs. **2.** 5 × 10^4^ ES cells + 10^4^ T cells stained with IFN- γ Ab or granzyme B monoclonal Ab. **Spheroid assay:** 2 × 10^3^ ES cells were put into sphere culture medium to assess the sensitivity of the spheres to lysis by E cells. The largest possible diameters of perpendicular spheres were measured.	**Cytotoxicity assay:** E group lysed ES cells at 1:1 and 0.5:1 E:T ratios. **Cytokine assay:** GD2 expression in many ES cells is sufficient to specifically activate T cells in E group to eliminate tumor cells by granzyme B secretion. Also, co-incubation of E group with the GD2^+^ ES cells induced production of IFN- γ and TNF-α. **Spheroid assay:** Sixteen-hour cocultures of Ewing sarcoma spheres with E group resulted in efficient lysis, whereas C group had no visible effect on sphere structure.

+: Were co-culture with.

Abbreviations: Ag: antigen, AM: calcein-acetyoxymethyl, att: Anchored Tumor Targeted, BsAb: bispecific antibody, CAR: chimeric antigen receptor, Ctrl: control, CD: cluster of differentiation, Cr: Chromium, CSV: cell-surface vimentin, CT: Chemotherapy, d: day, DMSO: dimethyl sulfoxide, DXR: doxorubicin, EAT: ex-vivo armed T-cell, ELISA: enzyme-linked immunosorbent assay, Epha2: ephrin type-A receptor 2, ES: Ewing sarcoma, E:T: effector-to-target, Eu: Europium, EZH2: Enhancer of Zeste Homolog 2, g: group, FasL: Fas ligand, G-CSF: Granulocyte colony stimulating factor, GD2: disialoganglioside 2, h: hour, HLA: Human leukocyte antigen, IFN-γ: interferon-γ, IGF1R: type I insulin-like growth factor receptor, IL: interleukin, LDH: Lactate dehydrogenase, NB: neuroblastoma, OS: osteosarcoma, ROR1: tyrosine kinase-like orphan receptor 1, Th: T helper, TDA: topological data analysis, TNF-α: tumor necrosis factor α, TRAIL: Tumor Necrosis Factor-Alpha-Related Apoptosis-Inducing Ligand, ttIL: tumor-targeted IL-12, UTD: un-transduced, VEGFR2: Vascular Endothelial Growth Factor Receptor 2, wtIL12: Wild-type interleukin 12.+: Were co-culture with.

Abbreviations: Ag: antigen, AM: calcein-acetyoxymethyl, att: Anchored Tumor Targeted, BsAb: bispecific antibody, CAR: chimeric antigen receptor, Ctrl: control, CD: cluster of differentiation, Cr: Chromium, CSV: cell-surface vimentin, CT: Chemotherapy, d: day, DMSO: dimethyl sulfoxide, DXR: doxorubicin, EAT: ex-vivo armed T-cell, ELISA: enzyme-linked immunosorbent assay, Epha2: ephrin type-A receptor 2, ES: Ewing sarcoma, E:T: effector-to-target, Eu: Europium, EZH2: Enhancer of Zeste Homolog 2, g: group, FasL: Fas ligand, G-CSF: Granulocyte colony stimulating factor, GD2: disialoganglioside 2, h: hour, HLA: Human leukocyte antigen, IFN-γ: interferon-γ, IGF1R: type I insulin-like growth factor receptor, IL: interleukin, LDH: Lactate dehydrogenase, NB: neuroblastoma, OS: osteosarcoma, ROR1: tyrosine kinase-like orphan receptor 1, Th: T helper, TDA: topological data analysis, TNF-α: tumor necrosis factor α, TRAIL: Tumor Necrosis Factor-Alpha-Related Apoptosis-Inducing Ligand, ttIL: tumor-targeted IL-12, UTD: un-transduced, VEGFR2: Vascular Endothelial Growth Factor Receptor 2, wtIL12: Wild-type interleukin 12.

^e^Different methods of gene transfer to T-cells.

a Specific lysis formula: [(test release − spontaneous release)/(maximum release − spontaneous release)] × 100.

b HLA-G and HLA-E are nonclassical HLA molecules with known immunosuppressive function

c GM-CSF production by CAR T cells to IL18 receptor signaling (GM18).

d Subtoxic concentrations of CTs: 2 µM carboplatin, 240 uM ifosfamide, 100 µM etoposide, and 10 µM DXR.

**Table 3:** The findings of in vivo studies conducted to evaluate the efficacy of chimeric antigen receptor (CAR) T-cell therapy in primary bone tumors.

Author, year	Animal model	Study design	Main outcomes
Mensali, 2022	(NOD).Cg-Prkdcscid Il2rgtm1Wjl/SzJ (NSG) female mice	**Antitumor model:** IT injection of 1 × 10 ^6^ tumor cells followed by iv injection of 1 × 10^7^ OSCARs C) Mock T cell **Localized model**: The case group received IP injection of 1 × 10 ^6^tumor cells followed by IP injection of 1 × 10^5^ T cells: E1) cells. C) control T cell **Systemic model**: The case group received IV injection	A 100-fold higher tumor growth was observed in the animals treated with mock T cells than in the OSCAR groups. Similar levels of OSCAR-1 and OSCAR-3 T cells were found in the tibia, spleen, collateral bone marrow, and in the peripheral blood.From these data we concluded that OSCAR T cells were efficient in controlling several tumor cell lines at different anatomical locations.
Zhang, 2022	NCG 6–8 w old mice	**Short-Term Experience** (14d)**:** Three of 4 subgroups received SC injection of tumor cells followed by IV Injection of T cells: E1: 5 × 10^6^ (LD) B7-H3 CAR T E2: 1 × 10^7^ (HD) B7-H3 CAR T C1: UTD T cell C2: PBS **Long-Term Experience (**80d)**:** Three of 4 subgroups received SC injection of tumor cells followed by IV Injection of 5 × 10^6^ T cells: E1: B7-H3 CAR-T cells C1: UTD T cell C2: PBS	**Short-Term Experience:** **Antitumor model:** tumor-suppressive effects was Significant on the 7th d^a^. Tumors in E1 and E2 disappeared or became very little since the 12th d^a^. Euthanizing the mice on the 19th d^a^ revealed that 2/5 mice in E1, and 3/5 mice in E2 were in CR. **TI**: E1 and E2 showed significant presence of CAR T cells in their tumoral tissue on the10th d^a^. **Long-Term Experience:** There was obvious tumor suppressive ability in E1, No tumor appeared in the mice in E1 within 80 days, and all the mice were healthy and alive.
Hu, 2022	CB17SC SCID 6–8 w old of both sexes mice	**TI and Antitumor model:** Two of three subgroups received SC injection of Tumor cells Followed by IV injection of 2.5 × 10^6^ CAR T cells: E1: No treatment E2: attIL12-T cells C: Control CAR T The production of IL-12 and IFNγ in tumors measured using ELISA in tumor lysates. **Antitumor model for large OS tumors:** three subgroups received engineered T cells as below: E1) B7H3 CAR-T cells alone E2) attIL12-armored B7H3 CAR-T C) T cells	**TI:** According to IF staining, T cells in E1 accumulated in tumors, while very few T cells in (C) were able to infiltrate tumors. Also in E1 T cells produce significantly higher levels of IL-12 and IFNγ suggesting attIL12-T cells not only penetrate OS tumors but also are activated in them. **Antitumor model:** Compared with the rapid tumor growth in E1 and (C), In E2 cell transfer completely eradicated tumors in three of five mice. **Antitumor model for large OS tumors:** E1 failed to eliminate large OS tumors. In E2 T cells inhibited the progression of large OS tumors. Also not only accumulated in the tumors but also produced high levels of NKG2D and IFNγ. showed that attIL12 synergize with CAR-T cells to enhance tumor targeting, penetration, and T-cell activation in refractory large solid tumors.
Prinzing, 2021	NSG, 8–10 w old of both sexes mice.	**Localized model**: The case group received IP injection of 1 × 10^6^ of tumor cells followed by IP injection of 1 × 10^5^ T cells: E1) DNMT3A KO EphA2 CAR T cells. C) control T cell **Systemic model**: The case group received IV injection of 2 × 10^6^ Followed by 1 × 10^6^ T cells: E1) DNMT3A KO HER2.ζ-CAR T cells C) control T cell	**Localized model**: Both C and E1 had initial antitumor activity but only E1 T cells maintained tumor control, resulting in a significant survival advantage (P<0.05). **Systemic model**: Despite tumors expansion in C, 5/10 mice in E1 showed CR and improvement in survival.
Park, 2021	BALB-Rag2 (BRG) Male 6–10 w old mice	Two subgroups received SC injection of 10^6^ tumor cells randomizely followed by IV Injection of 2 × 10^7^ CAR T cells into^c^	TI: assessed by BLI: IHC staining of human CD3, CD4, and CD8 T cells in PDXs: the in vivo efficacy of GD2-EATs strongly correlated with the TILs density; GD2-EATs showed substantially ↑ TILs compared with those armed with other BsAb formats and also ↑ survival.
Talbot, 2021	NSG, female 8-w old mice.	**Antitumor model**: All subgroups received IT injection of 10 ^6^ tumor cells followed by IV Injection of B7-H3-CAR T cells in 4 escalating doses: E1: 3 × 10^5^ (LD), E2: 1 × 10^6^ (ID) E3: 3 × 10 ^6^ (HID), E4: 1 × 10 ^7^ (HD) (C): 3 × 10 ^6^ A nonsignaling version of the B7-H3-CAR **TI model**: 3 × 10^6^ CAR T cell injection after 28 d	A dose-dependent antitumor activity of B7-H3 CAR T cells was observed. All mice in E2, E3, E4 initially had CR to CAR T cell injection. E1, C showed progression of cancer leading to hindlimb amputation at d 100. TI: B7-H3-CAR T cells trafficked to engrafted right tibial tumors on d 3 post implantation and more tibial expansion on the 4th^a^ compared to C(p < 0.01). B7-H3-CAR T cells persisted at the primary tumor site through 14 d^a^.
Lange, 2021	NSG 7–10 w old mice	**To compare CAR.GM18**^f^**T-cells with standard CAR T-cells, 2 xng model designed:** **Model 1:** Two subgroups received SC tumor Injection of 2 × 10^6^ cells followed by IV Injection of 10^5^ (LD) or 3 × 10^5^ (HD) of different CAR T cells: E1) EphA2-CAR T cells E2) EphA2-CAR.GM18 T-cells For long-Term survival both E1, E2 received an additional SC injection of tumor cells in 102 to 104 d^a^. **Model 2:** Two subgroups received IP Injection of 1 × 10^6^ tumor cells followed by IV Injection of 10^5^ (LD) and 3 × 10^5^ (HD) of different CAR T cells: E1) HER2-CAR E2) HER2-CAR.GM18 T-cells	**Model 1:** In E1, CR induction in 4/14 (29 %) mice at LD g and in 6/9 (66 %) mice at HD g was observed. T-cells only improved survival at HD g. In E2, CR induction in 14/15 (93 %) at LD g and in 10/10 (100 %) of HD g was observed.Also there was a significant survival at both HD and LD. Recurrences after CR only observed in E1 received LD. Improved antitumor activity of E2 cells at LD correlated with a significant greater peak expansion in comparison to E1. There was no significant difference between E1 and E2 at HD in regard to peak T-cell expansion and persistence. We observed increased expansion of T-cells in E2 at LD. Since E2 T-cell proliferation depends on antigen density, improved expansion is most likely due to the fact that the tumor to CAR T-cell ratio is higher at the lower cell dose. In long-term survivors, only 1/19 mice received E1 or E2 T-cell therapy, developed an EphA2-negative tumor. **Model 2:** In comparison of E2 and E1cells at two HD and LD, E2 had potent antitumor activity at both cell doses resulting in a significant survival advantage.
Park, 2020	BALB-Rag2, 6–10 w old male mice	SC injection of 2.5 × 10^6^ Tumor cells GD2 expression confirmed by IHC. Besides tumor cell line xenografts, 3 different PDXs, GD2 + and HER2 + were established followed by IV Injection of 2 × 10^7^ T- cells. Ctrl: no Tx, unarmed T cells, Ctrl EATs (T cells armed with GPA33-BsAb).	GD2-EATs and HER2-EATs ↓ multiple OS PDX tumors size, had ↑ anti‑tumor effect against OS PDXs without significant toxicity, and ↑ survival compared to negative Ctrls (P<0.0001). TI: IHC staining for human CD3, CD4, and CD8 T cells showed infiltration of EATs inside tumors. CR: Although high-dose GD2-EATs released more IL-2 and TNF-α compared to Ctrls, Th-1 cell cytokines (except IFN-γ) were not significantly ↑ after EATs injection. Only ↑ IFN-γ was seen in GD2-EAT-treated mice compared to Ctrls. Combination of ICI and GD2‑ or HER2‑BsAb armed T cell was not effective
Hsu, 2020	NSG 6–8 w old mice	**Localized model:** All 4 subgroups received SC Injection of 2.5 × 10^5^ Tumor cells (OS and ES model) followed by IT injection of 5 × 10^6^ or IV injection of 5 × 10^6^ (LD) or 2 × 10^7^ (HD) different types of T cells: E1: EphA2 CAR T cells E2: Δ-CAR T cells C1: PBS C2: UTD T cell **Systemic model:** All above subgroups received IV injection of 2.5 × 10^5^ tumor cells resulted in reproducible metastatic burden in both the lungs and livers 14–16 d^a^ followed by IV injection of LD of E1 T cells.	**Localized model:** **IT injection**: TS decreased in E1 compared to other groups (P<0.01) in both ES and OS model. In E1 we observed improve in survival out to a mean of 74 d and 88 d in the OS and ES models respectively. **IV injection:** **LD:** In E1.g any significant decrease in TS, nor survival extension was not observed in OS model. survival was extended 39d, but not tumor regression in ES model. **HD**: In E1 cause decrease in TS and extended survival in both OS and ES models. Significant differences (P<0.01) in TS between E1 and other groups was first seen in the ES model 15 d^a^. **Systemic model:** significant decrease in the number of visible metastatic nodules counted in both the lungs and livers was observed.
Wang, 2019	NSG 6–8 w old mice	Two of 3 Subgroups received IT Injection of 5 × 10^6^ Tumor cells, followed by IV injection of 1 × 10^7^ T cells: E1: CD166.BBζ CAR T cell C1: UTD T cell C2: PBS	**TI:** The intratumoral T cell infiltration in E1 was greater than C1. **TS**: E2 T cells efficiently decrease TS when compared to the control groups (C1, C2).
Majzner, 2019	Immunodeficient NSG 6–12 w old of both sexes mice	**Localized model**: Both subgroups for both models (ES and OS) received IT Injection of 10^5^ tumor cells followed by IV Injection of 10^6^ different types of T cells: E1) B7-H3 CAR-T cells C) CD19 CAR T cell **Systemic model:** The case group received IT Injection of 10^5^ OS tumor cells followed by IV injection of 10^6^ CAR T cells: E1) B7-H3 CAR-T cells C) No treatment	**Localized model**: E1 T cells mediated CR and tumor eradication of both OS and ES xng. E1 T cells improve survival compared to C in OS and ES xng. **Systemic model:** All mice in C died within 50 d of amputation whereas 9/10 mice in E1 g survived more than 5 months. Therefore, B7-H3 CAR T cells mediate activity against both established and metastatic OS xng.
Charan, 2019	NSG 6–8 w old (Jackson) mice	**Localized model:** *Single therapy:* All three subgroups received IT Injection of 1 × 10^5^ tumor cells and AMG102 with or without IV injection of 2 × 10^6^ T cells: E1) AMG102 alone E2) CAR T cell C) UTD *Combination therapy:* All four subgroups received IT injection of 1 × 10^5^ tumor cell followed by IV injection of 2 × 10^6^ T cells: E1) CAR T cell alone E2) UTD alone E3) CAR T cell with AMG102 E4) UTD with AMG102 **Systemic model:** *Single therapy*: All three subgroups received IV injection of 1 × 10^6^ tumor cell with or without IV injection of 2 × 10^6^ T cells: E1) AMG102 alone E2) CAR T cell C) UTD T cell. *Combination Therapy*: All three subgroups received 1 × 10^6^ tumor cell followed by IV injection of 2 × 10^6^ T cells: E1) GD2CAR T cell alone E2) AMG102 with GD2 CAR T cell C) UTD T cell	**Localized model:** *Single therapy:* mice in E1 showed moderately decrease in tumor burden in the bone and increased the survival of mice rec relative to other subgroups suggesting targeting of c-Met/HGF signaling extends survival rate without changing of TS. *Combination therapy:* most of the mice in E3 showed no sign of primary tumor development in the bone, and DFS was markedly extended. In our models in E3 there was a significant delay in tumor progression and combination treatment appeared to control/eradicate tumor effectively. **Systemic model:** *Single therapy:* the model designed to evaluate the drug effects on lung metastasis. E1 moderately decreased tumor burden in the lungs and increased the survival of E1 relative to others subgroups. *Combination therapy:*IVIS assessment of tumor burden performed at 50 days post-inoculation suggested markedly decreased tumor burden in the lungs of mice receiving E2 relative to E1 and C mice. Survival analysis revealed that nearly all mice receiving E1 developed lethal lung metastasis by 60–70 days, whereas most mice receiving E2 remained healthy until end of the study. Therefore, we observed, the effect of CAR-T cells or AMG102 as single agents had modest and not statistically significant.
Fernandez, 2017	NSG 10–12 w old mice	**IT model**: both subgroups of mice received IT Injection of 5 × 10^5^ Tumor cells with or without IT injection of 5 × 10^6^ cells: E1) UTD CD45RA^-^ cells E2) NKG2D-CAR T cells C) No treatment **IV model:** Rechallenge on d 60 was performed on survivor mice. Both subgroups received IV Injection of 1 × 10^5^ tumor cells followed by IV or IT injection of 5 × 10^6^ T cells: E1) IT injection of CD45RA^-^ NKG2DCAR^+^ cells E2) IV injection of CD45RA^-^ NKG2D-CAR^+^ cells C) No treatment	**IT model**: E2 mice (E:T ratio, 10:1) showed lower tumor burden, as seen with significantly lower average dorsal and ventral bioluminescent signals up to 56 d^a^. Tumor cells were only found in the lungs of mice E1,C. **IV model:** a significant extended survival time only for those mice receiving NKG2D CAR^+^ cells (120 days). All mice in (C) died from tumor burden within 2 weeks of the rechallenge (70.4 days for (C).
Long, 2016	Immunocompromised NSG 6–12 w old mice	To evaluate if ATRA can alter the quantity or suppressive phenotype of bone tumor-induced MDSCs, all subgroups received SC ATRA followed by SC or IT injection of 5 × 10^5^ or 10^6^ tumor cells. Subsequently they received IV injection of T cells: E1) GD2-CAR C) Mock T cells	increase in MDSC in sarcoma TM caused insignificant findings in vivo against 143b & EW8 tumor cells, (not due to the CAR T cell intrinsic factors d). In E1 increase in antitumor effect, decrease in TS and improve survival compared to C. ATRA treatment increased GD2 antigen expression on tumors upon exposure. Together, these results suggest that ATRA may improve efficacy of the GD2-CAR against sarcoma tumors through its effects on MDSCs.
Huang, 2015	NSG 6–12 w old with mixed gender mice	**Systemic model:** All 3 subgroups received IV injection of 7.5 × 10^5^ followed by IV Injection of 1 × 10^7^ T cells: E1) IGF1R CAR (IGZ) T cells E2) ROR1 CAR (RGZ) T cells C) mock T cells **Localized model**: Three of 4 subgroups received IP injection of 3 × 10^5^ followed by IV Injection of 1 × 10^7^ T cells: E1) IGF1R CAR (IGZ) T cells E2) ROR1 CAR (RGZ) T cells C) mock T cells	**Systemic model:** decrease in TS post CAR T cell injection (p < 0.05). improve survival in E2 compared to E1 g (p = 0.0261) but not against C g (p = 0.0864). Recurrent tumors were recovered from both CAR T cells. **Localized model**: decrease in TS post CAR T cell injection (p < 0.001). E2 T cells had no effect in TS compared to C g (p > 0.05). E2 T cells showed ani-tumoral activity (p < 0.01). E1 and E2 T cell improve survival (p < 0.05), but E1 T cells increase tumor suppressing capacity, and survival rate more than E2 (p < 0.01).
Liebsch, 2013	NOD/SCID 8–10 w old mice	Both subgroups received IV Injection of 2 × 10^6^ Tumor cells followed by IV injection of 1 × 10^7^ T cells: E1) 14.G2a-CD28z-transduced T cells C) analogous of UTD T cells	TS decreased in E1 g (P=0.0965). while at non-pulmonary sites indifferent TS was observed, E1 lead to lung tumor decrease in numbers and growth.WB-MRI revealed in vivo growth of tumors at all sites. No overall survival.
Kailayangiri, 2012	NOD/SCID 8–10 w old mice	Both subgroups received SC Injection of 5 × 10^6^ tumor cells followed by IT Injection of 5 doses of 10^7^ T cells: E1) 14.G2a-CD28z-transduced T cells C) UTD T cell	**TS:** TS at first Injection was similar in both groups, but over time in E1 g, TS significantly decreased.
Rainusso, 2012	NOD/ SCID 4 to 5 w old male mice	Both subgroups received IT Injection of 2 × 10^4^ tumor cells followed by IV injection of 1 × 10^6^ T cells: E1) HER2-CAR T cells C) non-specific T cells	No difference in TS was observed between E1 and C. However, TS may not adequately reflect the therapeutic effect in TICs. On the other hand, not only a significant decrease in the sarcosphere forming efficiency from E1Tcells was observed but also the number of lung metastasis in E1 was decreased.
Huang, 2011	athymic nude 6 w old mice	Both subgroups received IT injection of 2 × 10^5^ tumor cells followed by IV injection of 10^7^ T cells: E1) IL-11Ra-CAR T cells C) Control T cells	**TI:** T cells in E1 and C both were present in the tumor 3 d a. **Metastasis model:** 3/5 mice in E1 g had no visible metastases, while 5/5 mice in C had metastases. The N and size of the lung nodules and the lung weight were also decrease in E1.
Ahmed, 2008		**Localized model:** The case group received IP Injection of 2 × 10^6^ tumor cells followed by IP Injection of 10 × 10^6^ CAR T cells: E1) HER2 CAR T cell C) No treatment **Systemic model:** The case group received IV Injection of 2 × 10^6^ tumor cells followed by daily IV injections of 10 × 10^6^ CAR T cells: E1) HER2 CAR T cell C) No treatment	**Localized model:** E1 induced tumor regression in 9/10 mice bearing small tumors and 7/9 mice bearing large tumors.mice bearing small tumors had a median survival of > 160 days (*P*<0.001) whereas mice with large tumors had a median survival of 88 days (range 62 to > 160 days; *P*<0.01). **Systemic model:** In Cg, tumors progressively grew in all animals. In contrast, T cells in E1 resulted in rapid regression of lung metastasis in all treated animals. 8/10 animals had no evidence of tumor recurrence after > 6 months of follow-up. Survival analysis revealed a significant survival advantage (P<0.001) in E1 (median 145 days) compared to C (median 65 days)

Abbreviations: ATRA: all-trans retinoic acid, att-IL12: Anchored Tumor Targeted-IL12, BsAb: bispecific antibody, CR: complete response, C: control group, d: day, DFS: disease-free survival, DNMT3A KO: DNA methyltransferase 3 alpha knockout, ES: Ewing sarcoma, E: Experiment group, ELISA: enzyme-linked immunosorbent assay, E:T: effector to target, EAT: ex-vivo armed T cell, g: group, HD: high dose, HER: human epidermal growth factor receptor, HID: high intermediate dose, IHC: immunohistochemistry, IP: intraperitoneal, IT: intratibial, IV: intravenous, IGF1R: type I insulin-like growth factor receptor, IVIS: In Vivo Imaging System, ID: intermediate dose, IL:interleukin, LD: low dose,m: month, MDSC: myeloid-derived suppressor cells, NTD: non-transduced-T cells, NKG2D: natural killer group 2 member D, NOD: non-obese diabetic, NCG: NOD CRISPR Prkdc Il2r Gamma or triple-immunodeficient,OS: osteosarcoma, PBS: phosphate-buffered saline, PDX: patient-derived tumor xenograft w: week, WB-MRI: whole body magnetic resonance imaging, ROR1: tyrosine kinase-like orphan receptor 1, SCID: severe combined immunodeficiency, SC: subcutaneous, TS: tumor size, TI: tumor infiltration, TIC: target tumor-initiating cell, TM: tumor microenvironment, UTD: un-transduced T cell, xng: xenograft.

^b^MG63.3 was clonally derived from the MG63 cell line because of its propensity to metastasize to the lungs.

^e^NCG mice are triple immunodeficient and lack functional/mature T, B, and NK cells, and have reduced macrophage and dendritic cell function

^g^AMG102: (rilotumumab) a fully human monoclonal antibody against HGF that prevents interaction between HGF and c-Met.

a After injection.

c All animal experiments were repeated twice more with different donor’s T cells.

d T-cell exhaustion induced by antigen-independent signaling of GD2-specific CARs.

f GM-CSF production by CAR T-cells to IL18 receptor signaling.

### 2.2 Quality assessment

SYRCLE’s risk of bias was utilized for checking the quality of the animal studies, and the findings revealed that 19 cases had good quality and one case was of moderate quality (Table 4). For in vitro studies, a checklist was designed and showed 20 high and 3 moderate qualities (Table 5).

**Table 4:** Quality assessment of the selected in vivo studies based on SYRCLE’s RoB [82].

Author, year	Selection^a^			Performance^b^		Detection^c^		Attrition^d^	Reporting^e^	Other^f^	Overall quality
	1	2	3	4	5	6	7	8	9	10	
Mensali 2023	*	*	*	*	*	*	*	*	*	*	High
Zhang, 2022	*	*	*	*	*	*	*	*	*	*	High
Hu, 2022	*	*	−	*	*	*	*	*	*	*	High
Prinzing, 2021	*	*	*	*	−	*	*	*	*	*	High
Park, 2021	*	*	*	*	*	*	*	*	*	*	High
Talbot, 2021	*	*	*	*	*	*	*	*	*	*	High
Lange, 2021	−	*	−	*	*	*	*	−	−	*	Moderate
Park, 2020	−	*	*	*	*	*	*	*	*	*	High
Hsu, 2020	*	*	*	*	−	*	*	*	*	*	High
Wang, 2019	*	*	*	*	*	*	*	*	*	*	High
Charan, 2019	*	*	−	*	*	*	*	*	*	*	High
Fernandez, 2017	−	*	*	*	*	*	*	*	*	*	High
Long, 2016	*	−	*	*	*	*	*	*	*	*	High
Huang, 2015	*	*	−	*	*	*	*	*	*	*	High
Liebsch, 2013	*	*	*	*	*	*	*	*	*	*	High
Kailayangiri, 2019	−	*	*	*	−	*	*	*	−	*	High
Rainusso, 2012	*	*	*	*	−	*	*	*	*	*	High
Huang, 2011	*	*	−	*	*	*	*	*	−	*	High
Ahmed, 2008	*	*	*	*	*	*	*	−	−	*	High

Abbreviations: SYRCLE: SYstematic Review Centre for Laboratory animal Experimentation, RoB: risk of bias

^†^Items in agreement with the items in the Cochrane Risk of Bias tool.

a 1: The adequate generation and application of the allocation sequence ^†^, 2: Baseline similarity between groups or adjustment for confounders in the analysis, 3: Adequate concealing of the allocation^†^.

b 4: Random housing of the animals during the experiment, 5: Blinding of the caregivers and/or investigators from the intervention each animal received during the experiment.

c 6: Random animal selection for outcome assessment, 7: Blinding of the outcome assessor.

d 8: Adequate address of incomplete outcome data^†^.

e 9: Whether the study reports are free of selective outcome reporting^†^.

f 10: Whether the study reports are apparently free of other problems that could result in high risk of bias^†^.

* = Yes, – = No.

**Table 5:** Quality assessment of the selected in vitro studies.

**Author, year**	**Objective**^a^	**Design**^b^			**Cell culture and materials**^c^			**Assay methods**^d^			**Data collection**^e^			**Data analysis**^f^			**Overall quality**
	**1**	**2**	**3**	**4**	**5**	**6**	**7**	**8**	**9**	**10**	**11**	**12**	**13**	**14**	**15**	**16**	
**Hu, 2022**	*	−	*	*	*	*	−	*	*	*	*	−	*	*	*	*	High
**Wiebel, 2021**	*	*	*	−	*	*	*	−	*	*	*	*	−	*	*	*	High
**Long, 2021**	*	−	*	*	−	*	*	−	*	*	−	*	*	*	*	*	Moderate
**Talbot, 2021**	*	*	*	−	*	*	−	*	*	*	*	*	*	*	*	*	High
**Altvater, 2021**	*	*	*	*	*	*	*	*	*	*	*	*	*	*	−	*	High
**Lange, 2021**	*	−	−	*	*	−	*	*	*	*	*	*	*	*	*	*	Moderate
**Park, 2021**	−	*	*	*	*	*	*	*	*	*	*	*	*	*	*	*	High
**Park, 2020**	*	−	*	−	*	*	*	*	*	*	*	*	*	*	*	*	High
**Hsu, 2020**	*	*	*	−	−	*	*	*	*	*	*	*	*	*	*	*	High
**Englisch, 2020**	*	*	−	*	*	*	*	*	*	*	−	*	*	*	*	*	High
**Kailayangiri, 2019**	*	*	*	−	*	*	*	−	*	*	*	*	*	*	*	*	High
**Wang, 2019**	*	−	*	*	−	*	*	*	*	*	*	*	*	*	*	*	High
**Chulanetra, 2019**	*	*	−	*	−	*	*	*	*	*	*	*	*	*	−	*	High
**Charan, 2019**	*	*	*	−	*	*	*	−	*	*	*	*	*	*	*	−	High
**Fernandez, 2017**	*	*	−	−	*	*	*	*	*	*	*	*	*	*	*	*	High
**Long, 2016**	*	*	*	−	*	*	*	−	*	*	*	*	*	*	−	*	High
**Huang, 2015**	*	*	*	*	*	*	−	−	*	*	*	*	*	*	*	*	High
**Huang, 2012**	*	−	*	*	*	−	*	*	*	*	*	*	*	*	*	−	High
**Kailayangiri, 2012**	*	*	*	−	*	*	−	*	*	*	*	*	*	*	*	*	High
**Lehner, 2012**	*	−	*	−	*	*	*	*	*	*	*	*	*	*	−	*	High
**Ahmed, 2008**	*	−	−	*	*	*	−	*	*	*	*	*	*	*	−	*	Moderate

a 1: Clearly defined research question or objectives,

b 2: Detailed description of the experimental setup, 3: Proper control groups, 4: Replicates to ensure reliability,

c 5: Information on cell lines or primary cells used, 6: Source and authentication of cells, 7: Details about cell culture conditions,

d 8: Full description of the assays used, 9: Validation of assay methods, 10: Reproducibility of results,

e 11: Detailed data collection methods, 12: Instruments and equipment used, 13: Data collection time points,

f 14: Clear statistical methods and software used, 15: Presentation of raw data and statistical results, 16: Appropriate statistical tests,

* = Yes, – = N0.

## 3 Discussion

### 3.1 Primary bone tumor-associated antigens

The identification and exact targeting of the tumor, which minimizes damage to healthy tissue, is a vital stage in the production of CAR-T cells. The following antigens were evaluated as a potential target of CAR T cells in PBTs:

#### 3.1.1 Human epidermal growth factor receptor 2 (HER2)

HER2 is overexpressed in up to 60 % of primary osteosarcomas, causing aggressive disease and poor prognosis [77]. The HER receptor tyrosine kinase family (HER1-4) can signal as homo- and heterodimers when attached to epidermal growth factor, changing growth factor beta, and other ligands, propagating downstream oncogenic cascades [118]. Notably, HER2 lacks a cognate ligand but participates in aberrant signaling through heterodimerization [77]. Recent studies highlight that targeting HER2 could be a promising strategy for PBT treatment.

In a study by Rainusso et al., CAR T cells targeting HER2 were found to eliminate the growth and metastasis of chemo-resistant osteosarcoma cells that had high HER2 and CD133 expression [77]. They also demonstrated that HER2 CAR T cells have the potential to eradicate osteosarcoma tumor-initiating cells (TICs), using sarcosphere assays and orthotopic tumor models [77]. These findings suggest that incorporating HER2-targeted immunotherapy could help overcome drug resistance by eliminating TICs. According to Ahmed et al., HER2 CAR T cells increased survival by shrinking tumors in mice with localized tumors [116]. This led to rapid shrinkage of lung metastasis in all treated animals in the systemic model, and no recurrence was seen after 6 months [116]. The HER2 CAR-T cells without DNMT3A or GM18 in their structure also showed strong anti-tumor activity, as shown by improved survival rates in LM7 model with intraperitoneal locoregional osteosarcoma [57], [63]. These findings were further supported by clinical evidence.

In a phase I/II trial utilizing HER2-CAR T cells, Ahmed et al. observed that this approach has the potential to eradicate the tumor without causing dose-limiting side effects [72]. The study showed that 4 out of 19 subjects had stable disease, and one had 90 % tumor necrosis on histology after resection, resulting in an overall survival of 29 months for the cohort [72]. Collectively, these results substantiate HER2 as an emerging immunotherapy candidate to combat drug-resistant and metastatic osteosarcoma.

#### 3.1.2 Disialoganglioside (GD2)

GD2 is a surface glycolipid antigen that is only found in certain solid tumors, like osteosarcoma and Ewing sarcoma [119], [115]. A third-generation GD2-targeted CAR with CD28, OX40, and CD3z signaling domains was developed by Long et al. and was shown to kill GD2-positive sarcomas in vitro [115]. However, the accumulation of immunosuppressive myeloid cells hampered the anti-tumor effects in vivo [115]. Wiebel et al. noted that greater GD2 expression in osteosarcoma cells is associated with cell confluency, boosting the efficacy of GD2-specific CAR T cells in vitro [120]. Additionally, CAR T cells targeting GD2 were able to lower pulmonary metastasis and delay tumor growth in human Ewing sarcoma xenografts in mice [119], [74]. Noteworthy to mention, elevated PD-L1 on tumor cells can limit GD2-CAR T cell activity by stimulating PD-1-mediated T cell apoptosis [119]. Chulanetra et al. demonstrated that sub-therapeutic doxorubicin could synergistically enhance GD2-CAR T cell potency against osteosarcoma in vivo by alleviating cell cycle arrest [119], [74].

Altvater et al. found that HLA-G1 on myeloid bystander cells decreased GD2-specific CAR T cell degranulation, opposing Ewing sarcoma cells [121]. Charan et al. revealed that co-administration of GD2-CAR T cells with an HGF-neutralizing antibody effectively controlled both primary and metastatic Ewing sarcoma growth by overcoming deleterious c-Met/HGF signaling [70]. Meanwhile, epigenetic or genetic manipulations upregulating GD2 expression in Ewing sarcoma were found to improve GD2-CAR T cell therapy [122]. Finally, Park et al. confirmed bispecific antibody engagement of GD2 elicited T cell-mediated antitumor effects against osteosarcoma in vivo, further improved by PD-L1 checkpoint blockade [123]. These results suggest that GD2 could serve as an effective target for CAR T cell therapy for PBTs, enhancing its effectiveness through methods that surpass immune resistance within the tumor microenvironment.

#### 3.1.3 Natural killer group 2D (NKG2D)

The expression of NKG2D ligands on various tumor types and immunosuppressive cells inside TME makes them potential targets for cancer treatments [124]. Several immune cells, including CD8+T lymphocytes and natural killer (NK) cells, express NKG2D receptors [125]. NKG2D and NKG2D receptor interactions are necessary for NK-cell destruction of osteosarcoma TICs [126]. In recent years, the role of NKG2D and its ligands in malignancies has garnered more attention.

One of the main ligands for activating the immunological receptor NKG2D is MHC class I chain-related molecule A (MICA) [127]. In osteosarcoma, MICA expression was higher than in benign tumors and healthy bone tissue [128]. Lehner et al. demonstrated that NKG2D CAR T cells efficiently destroyed Ewing sarcoma cells that expressed NKG2D-L [129]. The use of mRNA transfection or lentiviral transduction to create CAR T cells contributed to higher surface expression of chNKG2D [129]. Nevertheless, the introduction of mRNA resulted in reduced ligand-triggered receptor down-regulation while also leading to elevated TNF-α secretion and enhanced Fas ligand expression in CAR T cells [129].

Lucía Fernandez et al. designed CAR T cells derived from CD45RA cells engineered to express an NKG2D, exhibited cytotoxicity against osteosarcoma cells, in vitro [130]. In an orthotopic osteosarcoma model, NKG2D-redirected memory CAR T cells succeeded in restricting tumor growth and extending longevity without evidence of toxicity [131]. The selectivity against malignant cells and safety profile substantiated the potential of retargeting memory T cell subsets towards NKG2D ligands frequently overexpressed in osteosarcomas and Ewing sarcomas [131]. Although more evidence is needed to assess the efficacy and safety of NKG2D-redirected CAR-T cell therapy, this approach has the potential to eradicate osteosarcoma and Ewing sarcoma cells.

#### 3.1.4 B7-H3 (CD276)

B7-H3 (CD276) is an immunologic checkpoint ligand aberrantly expressed across numerous solid tumor types, including osteosarcoma, associated with metastatic progression and poor prognosis [132]. This immunomodulator's surface-bound and soluble isoforms both contribute to immune evasion [132]. Specifically, B7-H3 triggers STAT3 signaling cascades in tumor cells, promoting proliferation and metastasis, while interacting with counter-receptors like TLT-2 on T cells antagonizes activation [133], [134]. This cancer-selective profile and function have spurred translational interest in targeting B7-H3 by CAR T cell therapy in solid tumors. Preclinical studies have shown that locally applying B7-H3-targeted CAR T cells could effectively kill orthotopic and metastatic tumors in a number of cases, such as pancreatic cancer and neuroblastoma, without causing any obvious side effects [135].

Biazzi et al. found that anti-B7-H3 CAR T cells have the potential to eradicate chordoma cells and prevent multicellular spheroid outgrowth [136]. In osteosarcoma, Majzner et al. revealed that B7-H3-redirected CAR T cells achieved dose-dependent ablation of disseminated tumors in vivo, eliminating lesions and prolonging survival using both local and systemic T cell administration in aggressive and immunosuppressive xenograft models [69]. It has also been shown that CD28 costimulatory domains in these constructs could lead to rapid proliferation while 4-1 BB had greater persistence, suggesting that aggressive tumors may require CD28 co-stimulation due to its rapid expansion, while tumors with a slow progress rate could be controlled with 4-1BB CAR-T cells [69].

Zhang et al. conducted in vitro cytotoxicity tests on osteosarcoma cell lines using third-generation CAR-T cells that specifically targeted the B7-H3 antigen, demonstrating a significant tumor-suppressive effect [137]. Furthermore, mice given intermediate high and high dosages of CAR T cells targeting B7-H3 in a metastasizing orthotopic model of osteosarcoma resulted in longer than six-month survival [138]. Initial results suggest that evaluating B7-H3 as a potential CAR target for osteosarcoma in more realistic models that replicate the clinical conditions of these tumors is warranted.

#### 3.1.5 Interleukin 11 receptor alpha subunit (IL-11Rα)

Interleukin-11 (IL-11) is an inflammatory cytokine implicated in osteosarcoma pathogenesis based on the aberrant overexpression of its cell surface cognate receptor, IL-11Rα, in tumor tissues [139]. Lewis et al. used an orthotopic model to show that osteosarcoma lesions in mice and humans had higher amounts of IL-11Rα than healthy bone-forming and lung areas [139]. Subsequent investigations by Huang et al. revealed that IL-11Rα-targeted CAR T cells could cause existing lung metastases of osteosarcoma to recede in vivo, reducing metastatic tumor burden and improving survival compared to controls [78]. An alternate strategy explored by Zhao et al. involved identifying a cyclic IL-11Rα binding peptide able to initiate cancer cell death upon receptor engagement through direct mitochondrial membrane disruption rather than immunogenic modulation [140]. Both approaches underscore the clinical relevance of disrupting IL-11/IL-11Rα signaling as an emerging therapeutic vulnerability in osteosarcoma.

#### 3.1.6 Ephrin type-A receptor 2 (EphA2)

EphA2 is a key receptor in Ephrin signaling that has a crucial function in embryonic growth and is also involved in different cancers, such as osteosarcoma and Ewing sarcoma [64]. Kenneth Hsu et al. tested the efficiency of CAR T cells that target EphA2 for the selective killing of human osteosarcoma and Ewing sarcoma tumor cells [64]. EphA2 CAR T cells demonstrated significant cytotoxicity to sarcoma cell lines that expressed EphA2 and produced more cytokines than control cells [64]. In animal experiments, they showed that EphA2 CAR T cells could induce cell death in tumor cells that expressed EphA2, leading to reduced tumor size and prolonged survival [64]. They also showed that EphA2-targeting CAR T cells could migrate and locate the metastatic tumor sites [64]. These outcomes indicate that EphA2-directed CAR T cells are specific and could be used as a potential target to eradicate Ewing sarcoma and osteosarcoma [64].

#### 3.1.7 Vascular endothelial growth factor receptor 2 (VEGFR2)

VEGFR2 is a vital membrane protein in endothelial cells, involved in the process of angiogenesis [141]. VEGFR2 is activated by the binding of VEGF ligands, which trigger intricate signaling pathways that regulate cell growth, movement, and survival [142]. In Ewing sarcoma, VEGFR2 has a critical role, as it is expressed in both endothelial and tumor cells of some patients [143], [144]. Preclinical investigations showed that VEGFR2-specific CAR T cells could penetrate solid tumors and inhibit tumor growth in several syngeneic mouse models [54]. The transfer of VEGFR2-redirected CAR T cells is currently being assessed in early-phase trials, including patients with solid cancers (NCT01218867) [54].

Englisch et al. created CAR T cells that target VEGFR2, a key protein in endothelial cells linked to Ewing sarcoma tumors [54]. When they encountered target cells, these CAR T cells had clear antigen-specific degranulation and cytokine generation [54]. Interestingly, the study showed that CARs with shorter or medium-length hinge domains had better functional performance [54]. The antigen-specific responses observed in this study demonstrate the potential of this approach and also emphasize the relevance of VEGFR2 as a therapeutic target in the complex scenario of Ewing sarcoma treatment.

#### 3.1.8 HLA-G and HLA-E

HLA-G and -E, in contrast to typical HLA molecules, are not involved in the presentation of peptides to T cell receptors and have little genetic variability [145]. With just seven isoforms (HLA-G1 to G7), HLA-G has a restricted polymorphism [146], [147]. These isoforms interact with inhibitory receptors expressed on T cells, NK cells, and myeloid cells, thereby reducing the multiplication and activities of effector cells [148], [149], [150]. HLA-E interacts with the inhibitory receptor NKG2A/CD94, which has a detrimental effect on the cytotoxic activity of CD8+T cells and NK cells [148], [149], [150]. Tumor cells frequently overexpress HLA-G and HLA-E, and inflammatory cytokines can induce expression [148], [149], [150]. Experimental data indicates that HLA-G and HLA-E play significant roles in immune escape in a variety of malignancies [148], [149], [150].

Altvater et al. found that while HLA-G1 on myeloid bystander cells reduces CART degranulation responses to Ewing sarcoma cells, HLA-G1 expression on endothelial stem cells does not directly inhibit cytolysis by GD2-specific CAR T cells [121]. Also the most human Ewing sarcoma biopsies showed HLA-E on tumor or infiltrating myeloid cells, induced in Ewing sarcoma cells by IFN-stimulation and GD2-specific CAR T cell therapy [121]. However, neither myeloid bystander cells nor Ewing sarcoma tumor cells could produce HLA-E to reduce the anticancer effector activities of CAR T cells [121]. This implies that antigen-specific T lymphocytes are mostly unaffected functionally by non-classical HLA molecules.

#### 3.1.9 Activated leukocyte cell Adhesion Molecule (ALCAM) CD166

Type I transmembrane protein CD166, also known as Activated Leukocyte Cell Adhesion Molecule (ALCAM), is from the immunoglobulin family [151]. It is thought that ALCAM is involved in several biological processes, such as hematopoiesis, inflammatory reactions, and neuronal outgrowth [152]. Prior research has indicated that it is linked to the development of many different cancers, including osteosarcoma [153], [154], [155], [156].

Wang et al. found that several osteosarcoma cell lines express CD166, and that CD166-targeted CAR-T cells integrated with 4-1BB cytotoxically treated osteosarcoma both in vitro and in vivo [157]. CD166-specific CAR-T cells eradicated osteosarcoma cells by the release of cytotoxic molecules like granzyme B and perforin [157]. The application of CD166-specific CAR-T cells causes a tangible drop in tumor growth and a concomitant extension of survival [157]. Moreover, the 4-1BB co-stimulation played a crucial role in fostering the generation of Th1 cytokines, pivotal for attracting antigen-presenting cells and augmenting the overall immune response [157]. These findings underscore the multifaceted potential of CD166-specific CAR-T cells in combating osteosarcoma, not only through direct cytotoxicity but also by leveraging co-stimulatory mechanisms to enhance T cell functionality [158]. This research contributes valuable insights to the ongoing optimization of CAR-T cell therapies, providing a foundation for further refinement and potential applications in the targeted treatment of osteosarcoma. However, the expression of CD166 by normal tissues might hinder the clinical translation of this approach.

#### 3.1.10 Receptor tyrosine kinase-like orphan receptor 1 (ROR1)

Tyrosine kinase-like orphan receptor 1 (ROR1) is a type I transmembrane protein [159]. Researchers have linked its overexpression to several malignancies, including B-CLL, mantle cell lymphoma (MCL), breast cancer, B-ALL, lung adenocarcinoma, melanoma, and ovarian cancer [159], [160], [161], [162], [163]. ROR1 participates in the migration and invasiveness of tumor cells [164], [165]. In recent years, ROR1 has gained significant attention as an immunotherapeutic target. Cirimtuzumab, a humanized monoclonal antibody that blocks ROR1 signaling, showed promise and safety in a phase I trial for individuals with relapsed, progressed, or unresponsive chronic lymphoblastic leukemia [166].

Huang et al. provided evidence that ROR1 exhibits significant expression levels in sarcoma cell lines, including Ewing sarcoma, osteosarcoma, rhabdomyosarcoma, and fibrosarcoma [44]. Transferring ROR1 and IGF1R CAR T cells from a patient with sarcoma also slowed down the growth of osteosarcoma xenograft models that were already set up and placed in NSG mice [44]. Furthermore, IGF1R and ROR1 CAR T cell infusion increased survival in a localized sarcoma model of NOD/SCID mice [44]. Their findings suggested that SB-modified CAR T cells can efficiently target both IGF1R and ROR1, and these CAR T cells have potential for treatment of high-risk sarcoma [44].

While Preclinical studies indicate that ROR1-targeted CAR-T cells are potentially effective in the treatment of osteosarcoma, the safety of ROR1-targeted CAR-T cells should be clearly evaluated. ROR1 expression has been found in normal human tissues, including the body and the gastric antrum [167], [168].

#### 3.1.11 Cell-surface vimentin (CSV)

Vimentin, a protein in the intermediate filament family, is essential for cell integrity and stress resistance [169]. However, overexpression of this protein in various epithelial malignancies, including prostate, gastrointestinal tract, central nervous system, breast, melanoma, and lung cancer, accelerates tumor growth and invasion, leading to a poor prognosis [169], [170], [171]. The CSV-targeted peptide VNTANST was fused before the stop codon of the IL-12 p40-encoding sequence to create tumor-targeted IL-12 (ttIL12) [169], [172]. The administration of ttIL12 therapy was demonstrated to be more effective than wtIL12 therapy in both murine and canine tumor models, resulting from the accumulation of ttIL12 protein at the tumors sites [173], [174].

Hu et al. studied the efficacy and security of intrathecal infusions of tumor-targeted T lymphocytes armed with TtIL-12 (attIL12) for the treatment of xenograft tumors generated from osteosarcoma patients [175]. The study demonstrated that attIL12-T cells selectively attacked tumor cells that expressed cell-surface vimentin [175]. This led to more effector T cells and more interferon γ generated in the tumor environment, leading to the increased number of dendritic cells and secondary T-cell responses [175]. Both attIL12- and aIL12-T-cell transfer removed the harmful effects of peripheral cytokine release [175]. This approach clarifies the secure use of IL-12-based T-cell therapy for bone tumors, as it can also enhance anticancer activity and lower the toxicity of already available T-cell therapies [95].

#### 3.1.12 Alkaline phosphatase-1 (ALPL-1)

The protein ALPL-1, also known as Alkaline Phosphatase Isoform 1, is a member of the alkaline phosphatase family, which contains enzymes that participate in dephosphorylation reactions [176]. Within primary bone tumors, particularly osteosarcoma (OS), ALPL-1 is widely and specifically localized to the outer layer of OS cells, including both primary and metastatic bone tumors [177], [178]. The identification of this target was achieved by the use of target protein antibodies (TP-1 and TP-3) that specifically react to ALPL-1, therefore defining it as a specific and applicable marker for osteosarcoma [179].

A higher level of ALPL-1 in osteosarcoma tissues, but its low cross-reactivity with other healthy tissues, especially those not associated with bone, confirms its significance as a useful therapeutic target [176]. Significantly, whereas serum alkaline phosphatase is frequently increased in bone disease, the distinct membrane location of ALPL-1 in osteosarcoma sets it apart from other isoforms and non-tumor tissues [176].

Employing CAR T cells to target ALPL-1 offers a highly promising approach because of its high tumor specificity and low off-target toxicity [53]. In preclinical settings, Mensali et al. that CAR T cells, which were modified to identify ALPL-1, exhibited significant cytotoxicity against OS cells [53]. Furthermore, these CAR T cells had not any adverse effects on healthy tissues such as normal stem cells [53]. Hence, ALPL-1 presents a convincing ratio between tumor specificity and safety, so allowing additional clinical exploration in CAR T-cell treatment for osteosarcoma [53].

### 3.2 Tumor microenvironment in the context of primary bone tumors

TME is an intricate ecosystem comprised of cells that coevolve with cancer cells and offer assistance during malignant transformation [180]. TME is composed of both cancerous and non-cancerous tumor cells, including cells derived from bone marrow, immune and inflammatory cells, endothelial and pericytes that constitute the tumor vasculature, cancer-associated fibroblasts (CAFs), and extracellular matrix (ECM) that interacts intricately with the tumor [181], [182]. These interactions lead to the production of growth factors, chemokines, and matrix-degrading enzymes, which aid in the growth and invasion of the tumor [183]. ECM is a multifaceted system made up of macromolecules with unique biomechanical, biochemical, and physical characteristics [184]. This system becomes dysregulated during the process of carcinogenesis, which promotes the development of a microenvironment that is conducive to tumor angiogenesis and inflammation [185]. Immunologic phenotypes and capacities of TME cells impact the course of the disease [185], [186].

To overcome the immunosuppressive TME, one approach is to create modified CAR-T cells that release immune-stimulating cytokines like IL-12, IL-18, or IL-15 [187], [188]. This modification helps to change the immunomodulatory environment, leading to improved survival of CAR-T cells [187]. It also promotes the recruitment of natural immune cells like T-memory cells and central-memory T cells, which are more suited to survival, and long-term presence [188].

Tregs essentially effect immune suppression within the TME by producing TGF-β, which reduces the effectiveness of immune cells [189]. Consequently, several methods have been created to block the TGF-β receptor on the outside of CAR-T cells. Using CAR-T cells with dominant negative receptors (DNRs) for TGF-β is one such tactic [188]. Furthermore, by activating the receptor that has been modified to convey signals via co-stimulatory domains, including 4-1BB or IL-12 stimulatory signals, swing receptors with chimeric signaling domains can change TGF-β signals [188]. Similarly, by joining the extracellular domain of IL-4 and the endo-domain of IL-7, cytokine receptors can transform inhibitory impulses into stimulating signals [190], [191]. The native TGF-β receptor-II (TGFBR2) gene in CAR-T cells was rendered inactive using the CRISPR/Cas9 technique, which increased the efficacy of CAR-T cells [192]. As a result, TGF-β activity was suppressed, which decreased the production of Treg cells and stopped the exhaustion of CAR-T cells [192].

TME is made up of tumor cells that support stromal cells, microvasculature, cytokines, and chemokines [193]. Tumor-associated macrophages, regulatory T cells, and Myeloid-derived suppressor cells (MDSCs) are examples of suppressive immunological components that are more prevalent in the TME than in normal tissues and can limit T cell activation [194]. Tumor-associated macrophages, regulatory T cells, and MDSCs are examples of suppressive immunological components that are more prevalent in the TME and can limit T cell activation [194]. Furthermore, cancer-associated fibroblasts (CAFs) generate an intercellular matrix on the outer edge of the TME and in the surrounding microvasculature, forming a physical barrier that prevents CAR-T cell penetration [194]. Both the “chemical barrier” and the “physical barrier” hinder the CAR-T cells’ ability to enter and invade, reducing their efficacy.

Tumor-associated macrophages (TAM) and T lymphocytes proliferate in the particular microenvironment of osteosarcoma due to its porous extracellular matrix and copious blood flow, which creates an immune-tolerant milieu that is conducive to tumor chemotaxis [194], [195]. One way to overcome this barrier is to use immune antibodies, like TAM, MDSC, and regulatory T-cell antibodies, to diminish suppressive immune elements [196]. Fourth-generation CAR-T cells are currently believed to regulate the TME because of their capability to release cytokines that enhance the immune response close to tumor cells [197].

Some studies suggest that increasing the number of immunological checkpoints on T cells with a combination of PD-L1 inhibitors will reduce immunosuppression [198]. Additionally, chemotherapeutic drugs have the ability to lower TME immune factor levels [199]. Targets such as FAP and CSPG4 that affect the microvasculature or tumor mesenchyme may also help CAR-T cells penetrate the TME [200]. Furthermore, local CAR-T cell injection could boost the cells' concentration and enhance their ability to infiltrate [201]. In addition, according to Li et al., employing porous microneedle patches during local injection may enhance infiltration even more without compromising activity, offering a path forward for useful therapeutic applications [202], [203].

T cells that have reached T-cell exhaustion have altered transcriptional patterns, including persistent overexpression of inhibitory receptors, as well as diminished effector activity [204], [205]. T-cell anergy is a common condition in TME and can work similarly to T-cell depletion, resulting in an inability to respond to antigens [205]. The main factors influencing CAR T-cell depletion in the TME are low oxygen and nutrition levels [206]. T cells are attentive to their metabolic surroundings, and research indicates that the metabolic state of the TME has a significant impact on T-cell efficacy [206]. It is probable that the same metabolic factors that decrease the immune system will also affect the efficacy of CAR T cells in primary bone tumors. Furthermore, large concentrations of cancer cells in tumors lead to an extreme increase in antigen stimulation [207]. As a result, CAR T cells are continuously activated by antigens, which influences the onset of fatigue [207], [208]. Research suggests that frequent exposure to antigens downregulates effector gene expression and inhibits mitochondrial oxidative phosphorylation, leading to epigenetic modifications [209]. These alterations in mitochondrial oxidative phosphorylation drive T cells towards terminal exhaustion and also influence mitochondrial depolarization [208]. Constant activation also causes CAR T cells to produce more PD-1, which interacts with PDL-1 on cancer cells, raising the risk of death and pushing the cells into fatigue [210]. Furthermore, metabolic reprogramming could be changed to cause T cells to become fatigued even when PDL-1 expression on cancer cells is not directly interacting with T cells [210], [211], [212]. Several pieces of evidence suggest that using less differentiated T cells to create CAR T-cells would lead to persistence in the TME as producing long-term memory response would be easier for these cells [213], [214].

### 3.3 Side effects

Developing effective strategies to reduce and manage side effects associated with CAR T cell therapy is crucial for the successful clinical translation of this approach. Preventing and addressing cytokine release syndrome (CRS), immunological effector cell-associated neurotoxicity syndrome (ICANS), and cytopenias are of the utmost importance in this regard [215].

Patients who receive CAR T cells may experience neurologic toxicities such as disorientation, delirium, aphasia, obtundation, myoclonus, and seizures [216]. The exact underlying mechanism causing these neurological side effects is still unknown [217]. Early identification of CRS is a key factor. In CAR T-cell, the time frame for onset of CRS is days to weeks after infusion, when T-cell expansion is at its peak [210]. The FDA recently approved tocilizumab for the treatment of severe or life-threatening CAR T-cell-induced CRS in adults and pediatric patients ≥2 years old [218]. Tocilizumab is a monoclonal antibody that competes with IL-6 to bind its receptor, IL-6R, thereby inhibiting IL-6 signaling in effector cells [210]. Steroids are the initial treatment for isolated ICANS [210]. Tocilizumab exhibits a limited ability to pass the blood–brain barrier, demonstrates restricted effectiveness in treating neurological toxicity, and has been linked to severity of neurotoxicity [219]. Therefore, the administration of tocilizumab should only occur in cases of ICANS in conjunction with ongoing CRS.[220].

CRS, caused by the fast activation of the immune system triggered by CAR-T cells, is the primary and most important complication associated with this treatment [221]. CRS presents with an initial fever and may develop into a dangerous condition characterized by capillary leakage, low oxygen saturation, and lower blood pressure [221]. The clinical manifestations of CRS are correlated with the activation of T cells and elevated levels of cytokines, such as IL-6 and IL-1 [222]. The use of Anakinra, an IL-1 receptor (IL-1R) antagonist that has shown success in CRS treatment, is one of the several medications that have been developed to prevent CRS [222]. Additionally, previous reports suggest that CAR-T cells secreting an IL-1R antagonist can mitigate CRS-related death [219]. Another strategy for treating CRS and neurotoxicity could be to neutralize granulocyte–macrophage colony-stimulating factor (GM-CSF), a significant monocyte activator [223]. A novel study has demonstrated that catecholamine blocking using atrial natriuretic peptide (ANP) can prevent the production of cytokines and catecholamines that arise from the interaction of CAR-T cells and tumors [224].

### 3.4 Cost considerations and practicality of CAR-T cells

Despite its potential, the cost-effectiveness of CAR T-cell therapy remains a subject of controversy due to its high price and the uncertainty surrounding the clinical evidence [225]. Beyond the significant cost associated with producing CAR T-cell therapy, the process and facility costs further intensify the financial burden, especially given the frequent occurrence of adverse events such as CRS. Compared to conventional treatments, clinical studies employed relatively brief follow-up periods [225]. Furthermore, there is a lack of head-to-head comparative effectiveness data, which is a crucial consideration when assessing the cost-effectiveness of a novel treatment [226]. Further evidence is required to mitigate the uncertainty surrounding the current clinical data to thoroughly assess the long-term effectiveness, safety, and comparative efficacy [226].

The FDA granted approval for the utilization of CAR T-cell therapy in patients diagnosed with relapsed or refractory (R/R) follicular lymphoma (FL) who have undergone at least two prior lines of therapy [227]. However, its cost-effectiveness in comparison to other contemporary therapeutic approaches for relapsed or refractory follicular lymphoma (R/R FL) remains uncertain [228]. Idecabtagene vicleucel (Ide-cel) and cilta-cel were found to have higher quality-adjusted life years (QALYs) compared to salvage chemotherapy, with Ide-cel showing an incremental increase of 1.19 QALYs and Cilta-cel showing an incremental increase of 3.31 QALYs [227]. The incremental costs associated with Ide-cel were US$140,693, resulting in an incremental cost-effectiveness ratio (ICER) of $118,229 per QALY [229]. Similarly, Cilta-cel had incremental costs of $119,806, leading to an ICER of $36,195 per QALY [229]. The difficulties of cross-trial comparison and the use of artificial or historical control arms have hampered many of these analyses, as all CAR T-cell treatments first received approval through single-arm clinical trials [230].

### 3.5 Strategies to enhance the efficacy of CAR T cell therapy

The application of CAR-T cell-based cell therapy in the treatment of hematological malignancies is an emerging field that has provided optimism to patients previously believed to be incurable. CAR T cell therapies aimed at solid tumors encounter substantial difficulties because of the scarcity, reduced persistence, and impaired functionality of T cells within the immunosuppressive TME [231], [232]. Prolonged exposure to antigens results in T cell fatigue and a transformation of CD8 + T cells into a condition resembling NK-like cells, consequently limiting their efficacy [233]. Furthermore, dose-limiting toxicities (DLTs), such as off-target effects when normal tissues with low amounts of the target antigen are erroneously targeted, limit the capacity to increase therapeutic doses [234]. In an attempt to address these problems, the implementation of regional delivery of CAR T cells directly to the tumor site, alongside immunological checkpoint inhibitors such PD-1 inhibition, was carried out [235]. It showed promising results by reducing systemic toxicity and achieving consistent clinical and metabolic responses in particular patients [235]. However, it is still crucial to improve the effectiveness of CAR T cells in solid tumors. Next-generation CAR T cells adopt various approaches to address key limitations in cancer immunotherapy [234].

Antigen escape, in which tumors downregulate or lose expression of the targeted antigen, is another challenge that could result in tumor recurrence. An effective approach is the dual administration of CAR T cells that targets distinct antigens. Applying this approach increases the probability of achieving effective elimination of tumors, even in cases when one antigen is no longer detectable [236]. Alternatively, it is possible to modify a single T cell by incorporating dual CAR constructions or a single CAR construct specifically engineered for recognition two distinct antigens. This novel dual or tandem CAR strategy allows T cells to specifically target cancers that express either one or both antigens, so it decreases the risk of tumor evasion and recurrence [236].

Optimizing CAR T cells functions can be accomplished through the use of a number of techniques. T cells Redirected for Universal Cytokine Killing (TRUCKs) are designed to secrete cytokines, such IL-12 or IL-18, through activation [237]. These cytokines play a crucial role in enhancing the overall antitumoral immune response by recruiting and activating additional immune cells within the TME [238]. Also switch receptors are designed for transforming inhibitory signals from the tumor, into stimulatory signals [239]. Furthermore, another targeted approach which minimizes the incidence of systemic adverse effects is orthogonal cytokine receptors like the orthogonal IL-2 receptor (oIL2R) [234]. These receptors are tailored to respond exclusively to specific cytokines by proliferating CAR T cell selectively [240].

Advanced approaches are required to mitigate the on-target/off-tumor toxicities associated with CAR T cell therapies, particularly when healthy and malignant cells display similar antigens. In the context of hematological malignancies, CAR T cells are used in combination with genetically modified hematopoietic stem cell transplantation (HSCT), where the stem cells lack the target antigen (for instance CD33 knock-out HSCT) [241]. Thereby, healthy cells from CAR T cell-mediated destruction will be safeguarded [241]. The synNotch receptor allows T cells to differentiate between normal and tumor cells through combinatorial antigen recognition [242]. In “AND-gated” circuits, activation of one receptor induces transcription of a second, guaranteeing that tumor cells expressing both antigens are targeted but normal cells are spared [243]. “NOT-gated” circuits include an inhibitory receptor, which prevents T cell activation when antigens unique to normal cells are recognized. [242], [244], [245]. However, this approach does not fully eliminate the risk of tumor relapse due to antigen escape, such as antigen loss or downregulation [246].

Additionally, gene editing and gene ablation techniques using highly specific, and effective tools have opened up new possibilities. In order to minimize the need for personalized T cell modifications, universal CAR-T cells were made through advanced genome editing techniques such as Zinc Finger Nucleases (ZFNs), transcription activator-like effector nucleases (TALENs), and CRISPR/Cas9 [247]. These tools can create universal off-the-shelf CAR T cells, are pre-manufactured, genetically modified T cells that can be used in multiple patients without the need for personalization. They target the native TCR to reduce graft-versus-host disease (GVHD) and native HLA to prevent graft rejection [247]. Engineered Zinc Finger Nucleases (ZFNs) eliminate the endogenous TCR, effectively lowering the risk of GVHD [248]. Meanwhile, in order to target and disrupt the TCRα and CD52 genes the TALEN technology was developed which led to production of UCART19, an allogeneic, off-the-shelf CAR T cell product designed to be TCR-negative [249]. The CRISPR/Cas9 system enables precise and efficient disruption of multiple gene loci, therefore can affect CAR T efficiency in various aspects [250].

CRISPR/Cas9 modalities allow for accurate elimination of native TCRs in T cells, therefore promoting the expression of transgenic receptors specific to malignancy. As a consequence, there is an enhanced and highly responsive anti-cancer reaction, which greatly enhances the efficiency of modified T cells in specifically targeting and eradicating cancer cells [247], [251]. Also, this novel approach facilitates the generation of allogeneic donor cells and highly effective effector T cells resistant to tumor-induced inhibitory pathways such as PD-1 and CTLA4 [247]. To optimize targeting of multiple genetic sites, a protocol incorporating several guide RNAs within a CAR lentiviral vector was employed. In conjunction with an adeno-associated virus (AAV) repair matrix, it was used to directly insert CAR-encoding DNA into the TCR alpha chain locus [252], [253]. This strategy yielded TCR-negative, CAR-positive T cells with enhanced potency compared to conventional lentivirally transduced CAR T cells, attributed to their more physiological, TCR-like regulation of CAR expression, promising outcomes in targeting solid tumors [247].

TCR-negative off-the-shelf CAR T cells face the challenge of rejection by the patient’s T cells due to HLA mismatches, leading to immune recognition and elimination. Lymphodepletion through chemotherapy or irradiation prior to CAR T infusion may delay this rejection until immune recovery [254]. The persistence of universal HLA-positive CAR T cells is expected to be limited [255]. Since prolonged CAR T cell persistence, as seen with CART19, has been linked to increased responses in multiple studies, early rejection of these cells could result in reduced and short-lived therapeutic responses [256], [257]. To address this, gene-editing technologies, such as beta-2-microglobulin and TCR knockout have been developed to generate HLA class I-negative CAR T cells, preventing rejection [250]. In the absence of TCR, HLA class I, and PD-1, triple gene-edited CAR T cells demonstrate decreased alloreactivity, preserve functional potency, and alleviate (GVHD) [258]. Enhanced resistance to apoptosis without compromising efficacy has been achieved by engineering Fas-resistant CAR T cells [252], [253]. Although the lack of HLA class I on allogeneic CAR T cells can inhibit T cell rejection, it may still result in detection and elimination by natural killer (NK) cells as a consequence of the “missing self” reaction [259]. To tackle this issue, the implementation of mandatory expression of non-classical HLA molecules, such HLA-E and HLA-G, has been proposed as a method to protect CAR T cells against death caused by NK-cells [260]. Additionally, recent approaches include the overexpression of Siglec-7 and Siglec-9 ligands to reduce NK-cell toxicity toward HLA-negative CAR T cells [261]. Making a bank of universal CAR T cells from HLA-homozygous donors is another way to reduce rejection [262]. Although gene-editing technologies show potential for developing universally reproducible CAR T cells, additional study is required to include them into expansion procedures of therapeutic quality. Thorough evaluation of both effectiveness and safety is also necessary in early-phase trials.

### 3.6 Strengths and limitations

This study had some strengths. This initial systematic review study investigated CAR T cells' efficacy in PBTs and specified the challenges and opportunities for improving the overall survival of patients with such difficult-to-manage conditions. However, our study had some limitations in the preparation and interpretation of findings. First, the number of investigated studies and patients was limited. Second, the quantitative data synthesis was not reachable to conclude the superiority of one target antigen over another. Moreover, further clinical trial studies with clinical reports of overall survival, tumor recurrence, and response to treatment are highly required.

### 3.7 Current state and future directions

CAR-engineered T-cell therapy presented tremendous promise for certain hematologic malignancies, leading to regulatory approvals targeting CD19-positive diseases [70]. However, the application of this approach for patients with solid tumors, such as PBTs, has proven challenging thus far. Impediments likely include a paucity of target antigens, dynamic neoantigen expression, curtailed engineered T cell fitness and tumor infiltration, and profound immunosuppressive mechanisms propagated by the bone TME [65].

This study has tried to systematically review the CAR construct designs, giving a methodical look at the potential of CAR-redirected T cell approaches against PBTs. Across evaluated antigens such as HER2, GD2, and VEGFR2, preclinical activity has been documented to varying extents in osteosarcoma and Ewing sarcoma models, providing impetus for continued investigation. However, pronounced therapeutic effects in patients have yet to be achieved, underscoring the likely need for combinatorial modalities to mitigate immunosuppressive mechanisms and further empower CAR effector cell function. The early-phase investigation analysis reveals promising antigen candidates and emphasizes the need for next-generation CAR T cells resistant to stromal inhibition to achieve clinical gains against bone malignancies. Emerging technologies and strategies hold promise for overcoming these challenges and expanding the applicability of CAR T-cell therapies.

## Availability of data and material

All the data utilized for this study is available in the published literature, and the curated Excel files related to this systematic review will be available upon reasonable request from the corresponding author.

## Funding

The authors did not receive any financial support for the research and authorship. The article processing charges (APCs) related to the publication of this study were supported by the corresponding author’s institution (School of Medicine, Royal College of Surgeons in Ireland, Dublin, County Dublin, Ireland).

## CRediT authorship contribution statement

**Atefeh Barzegari:** Writing – review & editing, Writing – original draft, Visualization, Validation, Resources, Project administration, Methodology, Investigation, Formal analysis, Data curation, Conceptualization. **Fateme Salemi:** Writing – review & editing, Writing – original draft, Visualization, Project administration, Methodology, Investigation, Formal analysis, Data curation, Conceptualization. **Amirhossein Kamyab:** Writing – review & editing, Writing – original draft, Visualization, Resources, Methodology, Formal analysis, Data curation. **Adarsh Aratikatla:** Writing – review & editing. **Negar Nejati:** Writing – review & editing, Writing – original draft, Software, Formal analysis, Data curation. **Mojgan Valizade:** Writing – review & editing, Writing – original draft. **Ehab Eltouny:** Writing – review & editing, Writing – original draft, Visualization, Validation, Methodology, Data curation, Conceptualization. **Alireza Ebrahimi:** Writing – review & editing, Writing – original draft, Visualization, Validation, Supervision, Project administration, Investigation, Data curation, Conceptualization.

## Declaration of competing interest

The authors declare that they have no known competing financial interests or personal relationships that could have appeared to influence the work reported in this paper.
