# Supplementary material for: The efficacy and applicability of chimeric antigen receptor (CAR) T cell-based regimens for primary bone tumors: A comprehensive review of current evidence
Source: J Bone Oncol. 2024 Sep 22;48:100635. doi: 10.1016/j.jbo.2024.100635 (PMC11460493; doi:10.1016/j.jbo.2024.100635)
Supplement: Supplementary Data 1 [file mmc1.pdf]

Supplementary Materials

Supplementary 1- Search strategies used for each database to find relevant studies.

Database	Search strategy
**PubMed**	("Neoplasms, Bone Tissue"[MeSH Terms] OR "Bony Tissue Neoplasm*"[Title/Abstract] OR "Bone Tissue Neoplasm*"[Title/Abstract] OR "Bone Neoplasia*"[Title/Abstract] OR "Bone Tomour*"[Title/Abstract] OR "Bone Tumor*"[Title/Abstract] OR "Bone Cancer*"[Title/Abstract] OR "Bone Malignancies"[Title/Abstract] OR "Bone Malignancy"[Title/Abstract] OR " Bone Neoplasm*"[Title/Abstract] OR "Ewing Tumor*"[Title/Abstract] OR "Ewing Sarcoma*" [Title/Abstract] OR "Ewings Tumor*"[Title/Abstract] OR "Ewing's Tumor*"[Title/Abstract] OR "Ewings Sarcoma*"[Title/Abstract] OR "Ewing's Sarcoma*"[Title/Abstract] OR "Sarcoma, Ewing"[MeSH Terms] OR "Juxtacortical Osteosarcoma*"[Title/Abstract] OR "Osteosarcoma, Juxtacortical"[MeSH Terms] OR "Osteoid Osteomas*"[Title/Abstract] OR "Osteoma, Osteoid"[MeSH Terms] OR Osteoma*[Title/Abstract] OR Osteoma[MeSH Terms] OR Osteochondromatoses[Title/Abstract] OR "Cartilaginous Exostosis"[Title/Abstract] OR "Cartilaginous Exostoses"[Title/Abstract] OR Chondrosteoma*[Title/Abstract] OR "Osteocartilaginous Exostosis"[Title/Abstract] OR "Osteocartilaginous Exostoses"[Title/Abstract] OR Osteochondroma*[Title/Abstract] OR Osteochondromatosis[MeSH Terms] OR Osteochondroma[MeSH Terms] OR "Giant Osteoid Osteoma*"[Title/Abstract] OR Osteoblastoma[MeSH Terms] OR Osteoblastoma*[Title/Abstract] OR "Giant Cell Tumor of Bone"[MeSH Terms] OR "Giant Cell Tumor of Bone"[Title/Abstract] OR "Ossifying Fibroma*"[Title/Abstract] OR "Fibroma, Ossifying"[MeSH Terms] OR Osteosarcoma[MeSH Terms] OR Osteosarcoma*[Title/Abstract] OR "Osteosarcoma Tumor*"[Title/Abstract] OR "Osteogenic Sarcoma*"[Title/Abstract]) AND (Immunotherapy[MeSH Terms] OR Immunotherapies[Title/Abstract] OR "adoptive T cell transfer"[Title/Abstract] OR "Chimeric Antigen Receptor"[Title/Abstract] OR "chimeric antigen receptor targeted immunotherapy"[Title/Abstract] OR "chimeric antigen receptor T cell therapy"[Title/Abstract] OR "Immunotherapy adoptive"[MeSH Terms] OR "Adoptive Immunotherapy"[Title/Abstract] OR "Adoptive Immunotherapies"[Title/Abstract] OR "Adoptive Cellular Immunotherapy"[Title/Abstract] OR "Adoptive Cellular Immunotherapies"[Title/Abstract] OR "CAR T-Cell*"[Title/Abstract] OR "CAR-T" [Title/Abstract] OR "CAR T"[Title/Abstract] OR "CAR T-Cell Therapy"[Title/Abstract] OR "CAR T-Cell Therapies"[Title/Abstract] OR "CAR T Cell Therapy"[Title/Abstract] OR "CAR T Cell Therapies"[Title/Abstract])
**SCOPUS**	((TITLE-ABS-KEY("Bony Tissue Neoplasm*") OR TITLE-ABS-KEY("Bone Tissue Neoplasm*") OR TITLE-ABS-KEY("Bone Neoplasia*") OR TITLE-ABS-KEY("Bone Tomour*") OR TITLE-ABS-

	KEY("Bone Tumor*") OR TITLE-ABS-KEY("Bone Cancer*") OR TITLE-ABS-KEY("Bone Malignancies") OR TITLE-ABS-KEY("Bone Malignancy") OR TITLE-ABS-KEY("Bone Neoplasm*") OR TITLE-ABS-KEY("Ewing Tumor*") OR TITLE-ABS-KEY("Ewing Sarcoma*") OR TITLE-ABS-KEY("Ewings Tumor*") OR TITLE-ABS-KEY("Ewing's Tumor*") OR TITLE-ABS-KEY("Ewings Sarcoma*") OR TITLE-ABS-KEY("Ewing's Sarcoma*") OR TITLE-ABS-KEY("Juxtacortical Osteosarcoma*") OR TITLE-ABS-KEY("Osteoid Osteomas*") OR TITLE-ABS-KEY(Osteoma*) OR TITLE-ABS-KEY(Osteochondromatoses) OR TITLE-ABS-KEY(Cartilaginous Exostosis") OR TITLE-ABS-KEY("Cartilaginous Exostoses") OR TITLE-ABS-KEY(Chondrosteoma*) OR TITLE-ABS-KEY("Osteocartilaginous Exostosis") OR TITLE-ABS-KEY("Cartilaginous Exostoses") OR TITLE-ABS-KEY(Osteochondroma*) OR TITLE-ABS-KEY("Giant Osteoid Osteoma*") OR TITLE-ABS-KEY(Osteoblastoma*) OR TITLE-ABS-KEY("Giant Cell Tumor of Bone") OR TITLE-ABS-KEY("Ossifying Fibroma*") OR TITLE-ABS-KEY(Osteosarcoma*) OR TITLE-ABS-KEY("Osteosarcoma Tumor*") OR TITLE-ABS-KEY("Osteogenic Sarcoma*"))) AND ((TITLE-ABS-KEY(Immunotherapies) OR TITLE-ABS-KEY("Chimeric Antigen Receptor") OR TITLE-ABS-KEY("adoptive T cell transfer") OR TITLE-ABS-KEY("chimeric antigen receptor targeted immunotherapy") OR TITLE-ABS-KEY("chimeric antigen receptor T cell therapy") OR TITLE-ABS-KEY("Adoptive Immunotherapy") OR TITLE-ABS-KEY("Adoptive Immunotherapies") OR TITLE-ABS-KEY("Adoptive Cellular Immunotherapy") OR TITLE-ABS-KEY("Adoptive Cellular Immunotherapies") OR TITLE-ABS-KEY("CAR T-Cell*") OR TITLE-ABS-KEY("CAR-T") OR TITLE-ABS-KEY("CAR T") OR TITLE-ABS-KEY("CAR T-Cell Therapy") OR TITLE-ABS-KEY("CAR T-Cell Therapies") OR TITLE-ABS-KEY("CAR T Cell Therapy") OR TITLE-ABS-KEY("CAR T Cell Therapies")))
**ISI web of science**	'Bony Tissue Neoplasm*") OR TS=("Bone Tissue Neoplasm*") OR TS=("Bone Neoplasia*") OR TS=("Bone Tomour*") OR TS=("Bone Tumor*") OR TS=("Bone Cancer*") OR TS=("Bone Malignancies") OR TS=("Bone Malignancy") OR TS=("Bone Neoplasm*") OR TS=("Ewing Tumor*") OR TS=("Ewing Sarcoma*") OR TS=("Ewing's Sarcoma*") OR TS=("Juxtacortical Osteosarcoma*") OR TS=("Osteoid Osteomas*") OR TS=(Osteoma*) OR TS=(Osteochondromatoses) OR TS=("Cartilaginous Exostosis") OR TS=("Cartilaginous Exostoses") OR TS=(Chondrosteoma*) OR TS=("Osteocartilaginous Exostosis") OR TS=("Osteocartilaginous Exostoses") OR TS=(Chondrosteoma*) OR TS=("Giant Osteoid Osteoma*") OR TS=(Osteoblastoma*) OR TS=("Giant Cell Tumor of Bone") OR TS=("Ossifying Fibroma*") OR TS=("Osteogenic Sarcoma*") OR TS=("Osteosarcoma Tumor*") OR TS=(Osteosarcoma*)) AND (TS=("Immunotherapies") OR TS=("Chimeric Antigen Receptor") OR TS=(adoptive T cell transfer) OR TS=("chimeric antigen receptor targeted immunotherapy") OR TS=("chimeric antigen receptor T cell therapy") OR

	TS=("Adoptive Immunotherapy") OR TS=("Adoptive Immunotherapies") OR TS=("CAR T-Cell*") OR TS=("CAR-T") OR TS=("CAR T") OR TS=("CAR T-Cell Therapy") OR TS=("CAR T-Cell Therapies") OR TS=("CAR T Cell Therapy") OR TS=("CAR T Cell Therapies"))

Supplementary 2- Attributions for the icons used in the graphical abstract.

1. Sick icons created by Freepik - Flaticon

2. Chemotherapy icons created by wanicon - Flaticon

 Radiation icons created by surang - Flaticon

Surgery icons created by Eucalyp - Flaticon

3. Cell icons created by Triangle Squad – Flaticon

 Antibodies icons created by Freepik - Flaticon

 Multiple myeloma icons created by HAJICON - Flaticon

4. Cell icons created by Icongeek26 - Flaticon

5. Rodent icons created by Freepik - Flaticon

Microscope icons created by justicon - Flaticon

6. Operation theater icons created by Three musketeers - Flaticon
